# Engineering Immune Cell to Counteract Aging and Aging‐Associated Diseases

**DOI:** 10.1002/advs.202521776

**Published:** 2026-01-28

**Authors:** Jianhua Guo, Lanjie Lei, Ying Jin, Lan Su, Shumao Cui, Liyun Shi

**Affiliations:** ^1^ Key lab of Artificial Organs and Computational Medicine Institute of Translational Medicine Zhejiang Shuren University Hangzhou Zhejiang China; ^2^ Zhejiang Cancer Hospital Hangzhou Institute of Medicine (HIM) Chinese Academy of Sciences Hangzhou China; ^3^ Department of Paediatrics & Adolescent Medicine Li Ka Shing Faculty of Medicine The University of Hong Kong Hong Kong SAR China; ^4^ State Key Laboratory of Food Science and Resources Jiangnan University Wuxi Jiangsu China

**Keywords:** aging, aging‐associated pathologies, cellular immunotherapies, clinical translation, mechanism exploration

## Abstract

Aging and age‐related diseases are a major public health concern, driving interest in anti‐aging research. While small molecules and natural compounds show promise in animals, clinical translation is limited. Recently, chimeric antigen receptor (CAR)‐engineered immune cells have achieved breakthroughs in treating non‐cancerous conditions like autoimmune diseases and organ fibrosis, highlighting their therapeutic potential. This review explains how the immune system counteracts aging through senescent cell clearance, reduction of pro‐inflammatory environments, and secretion of regenerative factors. It synthesizes principles of immune cell‐based anti‐aging therapies, analyzing preclinical and clinical studies. Key challenges include limited target specificity, immunosuppressive microenvironments, and variability in cell source and function. Future progress will require multidisciplinary collaboration—incorporating nanotechnology, synthetic biology, and targeted delivery—with artificial intelligence accelerating the development of personalized anti‐aging interventions. Cellular immunotherapies thus hold transformative potential for modulating aging and advancing precision medicine to extend global healthspan.

## Graphical abstract

This review highlights a paradigm shift in which advanced immune cell therapies, initially developed for cancer, are now being harnessed to combat aging. By engineering immune cells to selectively clear senescent cells and remodel pro‐inflammatory tissue microenvironments, these strategies offer a novel and powerful approach to delay age‐related functional decline and treat associated pathologies.

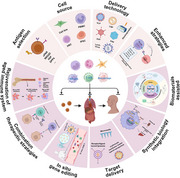

## 1 Introduction

Aging constitutes a biological phenomenon marked by the gradual deterioration of physiological functions over time. Fundamental mechanisms driving this process encompass cellular senescence, genomic instability, epigenetic alterations, chronic low‐grade inflammation (inflammaging), and age‐associated immune dysfunction, commonly referred to as immunosenescence [1, 2, 3, 4, 5, 6]. Senescent cells contribute to tissue damage by secreting the senescence‐associated secretory phenotype (SASP), which includes pro‐inflammatory cytokines such as interleukin‐6 (IL‐6) and tumor necrosis factor‐alpha (TNF‐α). This secretory profile facilitates the development of chronic pathologies, including malignancies [7], cardiovascular disorders [8], neurodegenerative diseases [9], metabolic syndromes [10], autoimmune conditions [11], and musculoskeletal degeneration [12, 13].

Current anti‐aging interventions encompass a variety of strategies, each characterized by distinct mechanisms and inherent limitations. Lifestyle modifications — such as photoprotection, consumption of antioxidant‐rich diets, and regular physical activity—have demonstrated modest efficacy in mitigating both cutaneous and systemic aging; however, their benefits necessitate sustained adherence and are subject to physiological limits. Pharmacological innovations, including senolytic agents like dasatinib combined with quercetin, which selectively target and eliminate senescent cells, exhibit therapeutic promise in contexts such as diabetic wound healing [10] Nonetheless, these agents face challenges related to off‐target toxicity and transient effects that require repeated administration. Senomorphic compounds, exemplified by rapamycin, aim to suppress SASP production but may inadvertently impair immune surveillance, thereby increasing vulnerability to infections and neoplasms [14]. Aesthetic treatments, including energy‐based devices, and injectable hyaluronic acid [15] or botulinum toxin [16], primarily address superficial manifestations of cutaneous aging without altering the fundamental biological aging processes. Advanced cellular and genetic therapies, notably mesenchymal stem cell (MSC) transplantation, have shown potential in enhancing tissue regeneration, such as myelin repair. However, their clinical application is constrained by suboptimal differentiation efficiency [17] and limited engraftment rates [18, 19]. Gene‐editing methodologies, for instance, exosome‐mediated delivery of miR‐302b to reverse cell cycle arrest, have achieved partial rejuvenation in murine models [20], though comprehensive evaluations of long‐term biosafety remain outstanding.

Immunosenescence refers to the progressive decline in immune system functionality, characterized by diminished production of immune cells, persistent low‐grade inflammation (inflammaging), and impaired immune memory. This phenomenon is intricately linked to a spectrum of age‐related diseases, including cancers, infectious illnesses, and neurodegenerative disorders [21, 22, 23, 24]. Recent progress in immune cell‐based anti‐aging therapies reflects a paradigm shift from broad‐spectrum clearance approaches toward precise immune reprogramming, garnering substantial scientific interest. These advances extend beyond the targeted elimination of senescent cells via engineered immune effectors such as CAR‐T [25, 26, 27], encompassing the development of “immune‐bridging” molecules through synthetic biology [28], the design of inflammation‐responsive CAR‐Macrophage (CAR‐M) [29], and the elucidation and modulation of immunosenescence signaling networks within organs, including the brain [30]. Collectively, this innovations offer promising therapeutic avenues for decelerating aging processes.

In this review, we concentrate on recent breakthroughs in immune cell‐based anti‐aging interventions and their implications for age‐associated pathologies, emphasizing the pivotal role of the immune system in fostering anti‐aging defenses and tissue repair. We further dissect the underlying scientific principles and strategic frameworks of these therapies, critically evaluating current challenges and limitations impeding clinical translation. Finally, we propose integrative solutions that combine senolytic strategies with immune rejuvenation technologies to enhance therapeutic efficacy.

## 2 The Role of the Immune System in Combating Aging and Aging‐Associated Diseases

The immune system functions not only as the primary defense against pathogens but has also been identified in recent research as a critical regulator of aging processes [31, 32, 33]. Key characteristics of aging include the accumulation of senescent cells, a decline in immune functionality, and the intensification of chronic inflammation. The immune system contributes to anti‐aging mechanisms through several interconnected pathways: (1) immune surveillance and removal of senescent cells; (2) mitigation of pro‐inflammatory microenvironments within tissues; and (3) promotion of tissue repair via regenerative cells and bioactive factors. Collectively, these integrated functions play a vital role in preserving organismal homeostasis by reducing the burden of cellular senescence, restoring immune balance, and decelerating age‐associated organ degeneration.

### 2.1 Immune Surveillance and Clearance of Senescent Cells

Senescent cells, defined by their irreversible cessation of cell division, serve as principal contributors to chronic tissue dysfunction and the aging process. Their detrimental impact is predominantly mediated through the senescence‐associated secretory phenotype (SASP), a pro‐inflammatory secretome composed of cytokines, chemokines, and proteolytic enzymes. This persistent inflammatory milieu disrupts tissue homeostasis and plays a significant role in the development of various age‐related pathologies [34, 35].

The pivotal function of immune cells in mitigating senescence is highlighted by accelerated aging models. Specifically, conditional deletion of the DNA repair gene Ercc1 in murine hematopoietic cells induces systemic immunosenescence, which subsequently promotes aging phenotypes across multiple non‐lymphoid tissues. This is evidenced by elevated oxidative DNA damage markers (such as 8‐oxoguanine), increased expression of senescence‐associated markers (P16^INK4a^ and P21^CIP1^), and pronounced tissue injury. Importantly, interventions including rapamycin administration or senolytic‐mediated elimination of senescent immune cells have been shown to delay these aging manifestations, underscoring the therapeutic potential of targeting this immune‐senescence axis [36].

Concurrently, the aging immune system itself undergoes functional decline, notably affecting critical effector populations such as natural killer (NK) cells. NK cells act as primary sentinels against senescent cells by recognizing and eradicating them through the activating receptor NKG2D, which binds to stress‐induced ligands (NKG2DLs) expressed on the surface of senescent cells, thereby triggering granzyme/perforin‐dependent cytotoxicity [37, 38]. Age‐associated impairment of this surveillance mechanism facilitates the accumulation of senescent cells. To restore this function, engineered approaches such as NKG2D CAR‐T cells have been developed. These therapies have demonstrated efficacy in reducing senescence biomarkers and enhancing physiological function in preclinical aging models, including non‐human primates, without eliciting significant toxicity [27]. Similarly, adoptive transfer of ex vivo‐expanded autologous NK cells has shown preliminary clinical promise by decreasing markers of senescence and inflammation in human subjects [39].

Beyond evading immune detection, senescent cells can actively establish an immunosuppressive microenvironment that promotes their persistence. A principal mechanism involves the upregulation of programmed death‐ligand 1 (PD‐L1) on p16‐positive senescent cells, such as alveolar macrophages and epithelial cells, within aged or inflamed tissues [40]. PD‐L1 engages programmed death‐1 (PD‐1) receptors on immune effector cells, thereby inhibiting their activity and facilitating senescent cell accumulation. Therapeutic targeting of PD‐L1 with monoclonal antibodies effectively eliminates p16^+^/PD‐L1^+^ senescent cells and reduces senescent cell burden in models of aging and chronic pulmonary disease [40]. To improve specificity for senescent cells, future strategies may integrate PD‐L1 targeting with additional senescence‐specific surface markers.

**FIGURE 1:**
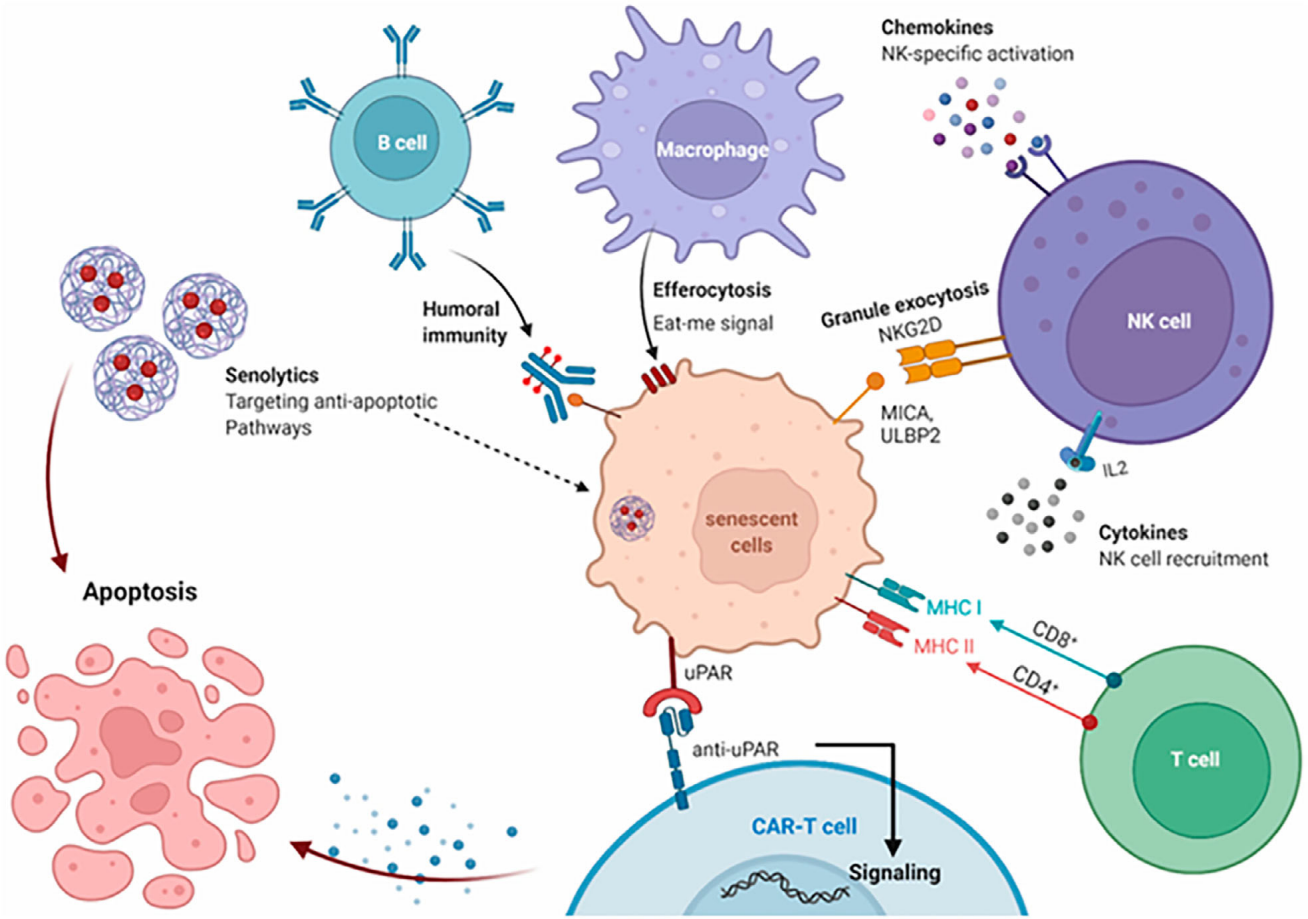
Clearance of senescent cells through immune system activation or apoptosis induction via senolytics. Reproduced with permission [42]. Copyright 2023, MDPI.

Recent advancements in therapeutic strategies have concentrated on the development of highly specific “search‐and‐destroy” mechanisms targeting senescent cells (see Figure 1). A key molecular target in this context is the urokinase‐type plasminogen activator receptor (uPAR), which is significantly upregulated on senescent cells across diverse biological settings [25, 26, 41]. uPAR‐targeted CAR‐T cell therapy has demonstrated substantial efficacy, achieving sustained clearance of senescent cells, reversal of insulin resistance, mitigation of hepatic fibrosis, and extension of healthspan in models of natural aging and metabolic disorders [25]. Expanding beyond cytotoxic modalities, uPAR‐directed CAR‐M represent an innovative approach that exploits the phagocytic and tissue‐remodeling functions of macrophages. These engineered macrophages not only engulf uPAR‐expressing senescent cells, such as profibrotic hepatic stellate cells, but also actively degrade fibrotic extracellular matrix components and recruit endogenous T cells to potentiate the anti‐senescence immune response. This multifaceted therapeutic strategy holds promise for the treatment of fibrotic diseases, including liver fibrosis [26].

### 2.2 Amelioration of Inflammatory Microenvironments

The persistent low‐grade inflammatory state characteristic of aging serves as a critical mechanistic contributor to the decline in tissue functionality and the development of various diseases. This inflammatory milieu is driven by aged and dysfunctional immune cells that adopt pro‐inflammatory phenotypes. For example, in the context of metabolic aging, senescent adipose tissue macrophages (ATMs) tend to polarize toward a pro‐inflammatory M1‐like phenotype, secreting tumor necrosis factor‐alpha (TNF‐α), which disrupts adiponectin signaling and fosters systemic insulin resistance [43, 44]. Similarly, within the central nervous system, senescent microglia release interleukin‐1 beta (IL‐1β), which induces neuronal apoptosis and impairs oligodendrocyte function, thereby accelerating the pathological progression of Alzheimer's disease [45, 46].

Innovative therapeutic approaches are increasingly focused on utilizing engineered immune cells to actively resolve aberrant inflammation and restore tissue homeostasis, marking a paradigm shift from broad immunosuppression to precise immunomodulation. A prominent strategy involves the direct reprogramming of key inflammatory effector cells. Specifically, engineered interventions aim to convert senescent, pro‐inflammatory M1‐like macrophages into regenerative, anti‐inflammatory M2‐like phenotypes. Notably, advanced inflammation‐sensing CAR‐M have been developed to perform therapeutic functions selectively within inflammatory microenvironments. These cells detect the principal inflammatory mediator TNF and subsequently activate an intracellular interleukin‐4 (IL‐4) signaling cascade, thereby programming macrophages toward an anti‐inflammatory phenotype [29]. Parallel approaches exploit the intrinsic regulatory capabilities of mesenchymal stem cells (MSCs), which suppress pro‐inflammatory cytokine production through secreted factors such as prostaglandin E2 (PGE2) and interleukin‐10 (IL‐10). Additionally, MSCs can deliver microRNAs (e.g., miR‐146a) via exosomes to target myeloid cells, inhibiting their nuclear factor kappa‐light‐chain‐enhancer of activated B cells (NF‐κB)‐mediated inflammatory pathways [49, 51]. This targeted reprogramming strategy is more nuanced than mere cellular clearance, as it aims to restore the protective functions of these immune cells.

Aging perturbs both the generative capacity and intercellular communication within the immune system, prompting advanced engineering efforts to restore immune homeostasis. One such approach involves the selective depletion of aged, inflammation‐prone hematopoietic stem cell (HSC) subsets — such as myeloid‐biased HSCs characterized by CD150 and CD47 expression — to rebalance lymphoid and myeloid lineage output and attenuate systemic inflammation [47]. Concurrently, enhancing immune cell longevity and intercellular signaling is essential. Emerging techniques include the use of engineered antigen‐presenting cells (APCs) to transfer telomeric sequences to T cells, thereby mitigating age‐associated telomere shortening and preserving immunological memory [48]. Moreover, exosome‐mediated delivery of regulatory molecules (e.g., miR‐21a‐5p) can promote the expansion of regulatory T cell (Treg) populations, reinforcing systemic anti‐inflammatory tolerance [49].

The therapeutic efficacy of cellular engineering is further augmented by concurrently targeting the dysfunctional metabolic and epigenetic landscapes of aged immune cells. For instance, supplementation with nicotinamide adenine dinucleotide (NAD^+^) precursors such as nicotinamide mononucleotide (NMN) enhances mitochondrial function and suppresses SASP in target cells [50]. Integrating such metabolic support within engineered cell designs fosters the development of therapies that maintain resilience within the aged tissue microenvironment.

Together, these engineered strategies—comprising the reprogramming of inflammatory cells, the restoration of immune homeostasis, and the provision of metabolic support—form a complex, synergistic framework. For instance, a therapeutic approach may integrate CAR‐modified macrophages engineered to phagocytose senescent cells with an exosome‐based payload aimed at reprogramming adjacent macrophages toward a reparative phenotype, concurrently administering systemic NAD^+^ supplementation to augment the longevity and functionality of both the therapeutic cells and the host immune system.

### 2.3 Facilitation of Tissue Repair

Beyond its canonical roles in surveillance and maintaining homeostasis, the immune system assumes a proactive and essential function in orchestrating tissue repair and regeneration following injury or during the aging process. This role is mediated by specialized subsets of immune cells that directly enhance stem cell activity, resolve fibrotic responses, and modulate the local microenvironment to facilitate effective healing.

**FIGURE 2:**
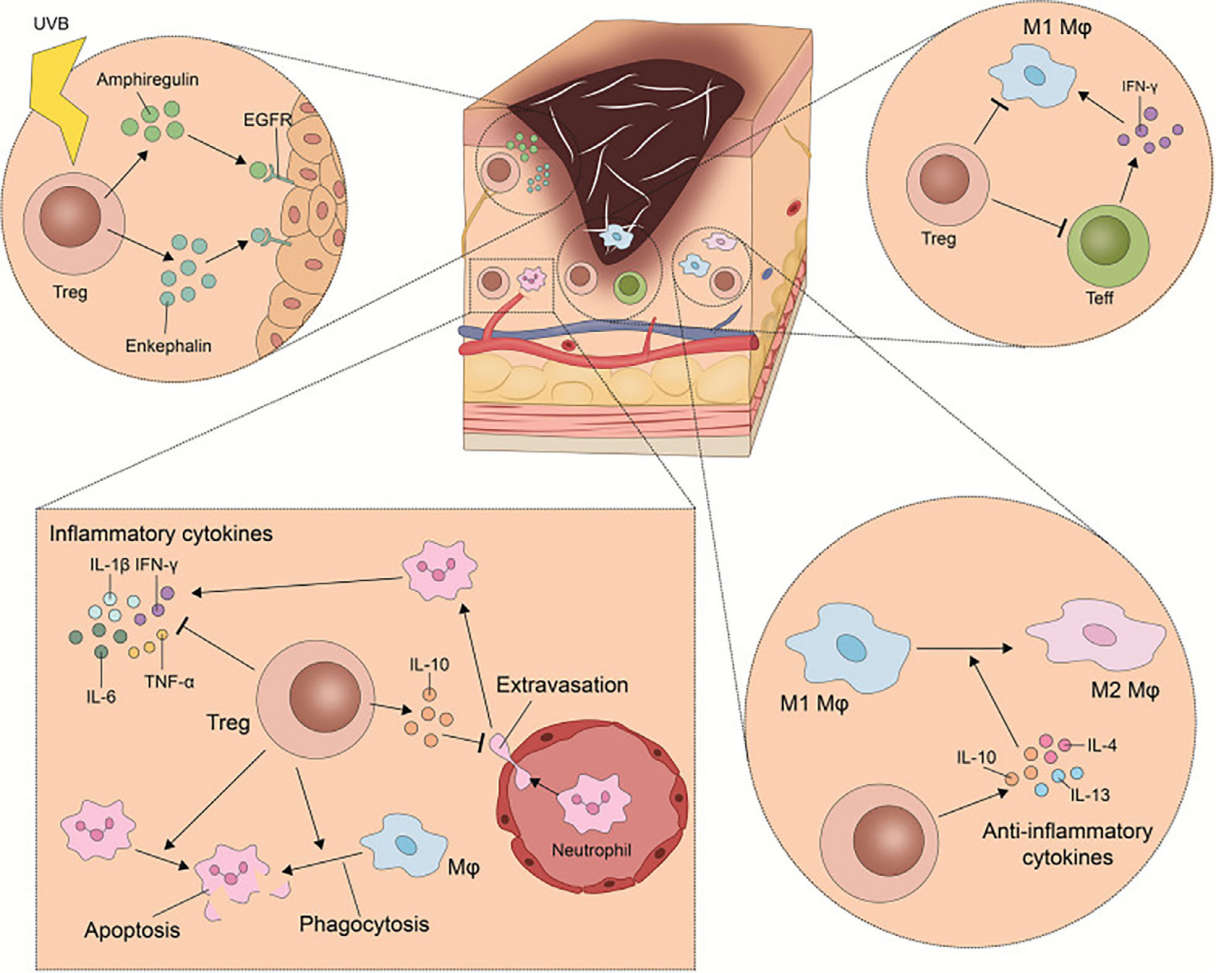
Cellular cross‐talks of Tregs in the skin. Reproduced with permission [51]. Copyright 2023, Springer Nature.

A notable example of this reparative capacity is demonstrated by Tregs (see Figure 2). In models of skin injury, Tregs are recruited to the wound site where they stimulate hair follicle stem cell proliferation through the expression of the Notch ligand Jagged‐1. Additionally, Tregs promote keratinocyte regeneration by secreting growth factors such as amphiregulin (AREG) and contribute to optimal wound healing by inhibiting pro‐fibrotic signaling pathways, including the IL‐17–CXCL5 axis, thereby mitigating excessive scar formation [51]. This exemplifies a precise mechanism whereby immune cells directly interact with tissue‐resident stem cells to coordinate regenerative processes.

More broadly, the immune system regulates tissue stem cell populations via a complex network of secreted cytokines and mediators. Immune cells residing within niches such as the bone marrow and skin secrete factors including interleukin‐2 (IL‐2), interleukin‐7 (IL‐7), and stem cell factor (SCF), which are critical for promoting stem cell proliferation and lineage specification [52]. Furthermore, immunomodulatory molecules such as TGF‐β and PGE2, along with exosomes containing microRNAs and proteins, modulate the regenerative potential of stem cells through paracrine signaling, thereby fine‐tuning the repair response.

Crucially, the restoration of an immune equilibrium is fundamental to sustaining these reparative functions. Aging is characterized by a shift in HSC differentiation bias toward the myeloid lineage, driven by a senescent and pro‐inflammatory microenvironment. This shift results in the accumulation of immunosuppressive populations, including myeloid‐derived suppressor cells (MDSCs), which impair tissue repair capacity [47, 53]. Senescent immune cells and MDSCs secrete factors such as TGF‐β that exert deleterious effects, for example, by activating the SMAD3 signaling pathway in MSCs and disrupting osteogenesis [53, 54, 55]. Consequently, therapeutic interventions aimed at rebalancing the aged immune system —such as the selective depletion of senescent or lineage‐biased HSCs — are critical for re‐establishing a microenvironment conducive to tissue regeneration.

Finally, the immune system's role in tissue repair intersects with metabolic reprogramming processes that underpin regeneration. Specific metabolites influence stem cell fate decisions and tissue repair; for instance, uridine enhances muscle and cardiac regeneration via the polyamine/pyrimidine metabolic axis [56], whereas glycolysis‐derived metabolites such as acetyl‐CoA and lactate facilitate stem cell reprogramming through epigenetic modifications including histone acetylation and lactylation [57]. Immune cells actively modulate this metabolic milieu, establishing a synergistic feedback loop that supports tissue renewal.

In summary, the immune system facilitates tissue repair through a multifaceted strategy encompassing direct cellular interactions (e.g., Tregs engaging stem cells), paracrine signaling of growth and regulatory factors, systemic rebalancing of immune cell populations, and integration with metabolic pathways. Leveraging these coordinated mechanisms holds significant promise for advancing regenerative medicine.

## 3 Strategies of Anti‐Aging Cellular Immunotherapy

Senescent cells display distinctive molecular and structural characteristics during the processes of aging and disease progression, which serve as essential biological bases for the development of targeted clearance approaches. First, alterations in membrane lipid composition—marked by decreased phospholipid levels, an increased cholesterol‐to‐phospholipid ratio, and reduced unsaturated fatty acid content—result in impaired membrane fluidity and permeability [58]. Simultaneously, lipid peroxidation induced by reactive oxygen species (ROS) further compromises membrane integrity [58, 59]. Second, senescence‐associated surface markers, such as heightened lysosomal β‐galactosidase (SA‐β‐gal) activity, overexpression of cyclin‐dependent kinase inhibitors(p16^INK4a^ and p21^CIP1^), and abnormal immunoglobulin (IgG) accumulation in the microenvironment [60], facilitate precise detection through proximity‐based fluorescent probes [61] or immunohistochemical methods. Moreover, senescent cells exhibit diminished signaling responsiveness via surface receptors (e.g., epidermal growth factor receptor, EGFR), contrasting with the SASP, which promotes the secretion of pro‐inflammatory cytokines (e.g., IL‐6 and IL‐8) and upregulates anti‐apoptotic BCL‐2 family proteins to evade programmed cell death [62, 63]. Morphologically, these cells are characterized by abnormal cell volume — resulting from dehydration or metabolite accumulation — and nuclear membrane invaginations [64]. From a metabolic perspective, mitochondrial dysfunction, evidenced by reduced mitochondrial number and loss of membrane potential, acts synergistically with lysosomal hyperactivation to exacerbate disruptions in energy metabolism [64].

Immunotherapeutic strategies employing immune cells have demonstrated significant efficacy in oncological research, particularly in the treatment of hematological malignancies [65]. Several critical factors ‐ including optimization of target selection, determination of cell sources, application of gene editing technologies, and implementation of combination strategies, collectively influence therapeutic outcomes. These fundamental considerations, extensively studied within cancer research, hold considerable promise for translation to the nascent field of anti‐aging interventions.

### 3.1 Antigen Selection

The identification of senescence‐specific cell surface markers has yielded critical therapeutic targets for anti‐aging strategies (see Table 1). Among this, uPAR is highly prioritized due to its marked upregulation in senescent cells and its strong linkage to the inflammatory microenvironment [41]. For example, CAR‐T therapy directed against uPAR enables the selective elimination of senescent cells, resulting in enhanced metabolic function and physical performance in aged mice. Notably, a single‐dose administration confers protective effects lasting up to one year [41]. Similarly, NKG2D‐CAR‐T therapy developed by an independent research group selectively targets senescent cells in animal models, extending lifespan by 20% to 30% and mitigating pathological damage induced by radiation or senescence [27]. The heterogeneity of NKG2D ligands — including MICA, MICB, and ULBP1‐6 — provides opportunities for multi‐target combinatorial therapeutic approaches.

**TABLE 1:** Potential biomarkers of senescent cell.

Protein Category	Target	Cell Type	Specificity	Key Considerations/ Limitations in Anti‐aging
Urokinase‐type plasminogen activator receptor	uPAR	multiple	High [25, 41]	Partial expression in activated immune cells and normal cells during migration/repair. Monotherapy targeting may cause off‐target effects, impairing immune function and wound healing
NKG2D ligands	MICA\MICB\ULBP1‐6	multiple	High [27]	Often overexpressed by tumor cells for immune evasion. Proteolytic shedding of these ligands by senescent cells can generate soluble forms that inhibit immune recognition, potentially promoting immune escape and chronic inflammation.
Surface receptors (subunits)	IL‐23R	multiple	Middle [66]	broad expression patterns in all nucleated cells, as standalone targets, specificity is unacceptably low, risking severe systemic side effects. Requiring integration with strict "AND‐gate" logic or or localized delivery.
	β‐2M	multiple	Middle [76]	
	CD97	multiple	Middle [77]	
Surface glycoproteins	CD44	multiple	Middle [78]	Systemic inhibition or targeting could disrupt glucose metabolism (DPP4) and immune surveillance (CD44). GPNMB is also overexpressed in malignancies such as melanoma.
	DPP4	multiple	Middle [73, 79]	
	GPNMB	multiple	Middle [80]	
Matrix‐associated proteins	FAP	fibroblasts	High [74]	FAP is also expressed on activated fibroblasts during physiological processes like wound healing. PDGFRβ is critical for vascular development and stability. Targeting these proteins may interfere with normal tissue repair and vascular homeostasis.
	PDGFRβ	Fibroblasts∖ pericytes∖ myofibroblasts	High [75]	

*Note*: “Middle” represents elevated in senescent cells, but also expressed in other cell types; “High” represents highly specific to senescent cells with minimal to no expression in other cell types.

Emerging targets include the interleukin‐23 receptor (IL‐23R), which exhibits elevated expression in senescent cells and demonstrates a positive correlation with systemic senescent burden [66]. IL‐23R may modulate inter‐organ inflammatory signaling pathways, thereby serving as both a biomarker and a therapeutic target. Additionally, cell surface glycoproteins such as CD44 influence immune recognition by modulating the adhesion of senescent cells to the extracellular matrix [67]. β‐2‐microglobulin (β‐2 M), the light chain component of the major histocompatibility complex class I (MHC‐I) involved in antigen presentation, is significantly elevated in its free form within senescent cells. This free β‐2 M is capable of crossing the blood‐brain barrier, entering the central nervous system, and impairing synaptic function through inhibition of N‐methyl‐D‐aspartate (NMDA) receptors, thereby contributing to cognitive decline [68, 69]. Furthermore, senescent hepatic cells secrete TGF‐β, which upregulates β‐2 M expression [54]. Subsequently, β‐2 M disseminates systemically via the circulation system to distal organs such as the kidneys and brain, inducing increased expression of p21 and promoting fibrotic process [54, 69]. Consequently, circulating β‐2 M levels represent a non‐invasive biomarker for assessing systemic senescent burden, with potential applications in evaluating overall aging and neurodegenerative disorders; however, interpretation must account for renal insufficiency, given the kidney's primary role in its β‐2 M metabolism. Separately, Glycoprotein Non‐Metastatic Melanoma Protein B (GPNMB), which is activated by oxidative stressors such as the lipid peroxide 4‐hydroxynonenal (4‐HNE), exhibits a twofold to threefold upregulation in senescent hepatocytes and neurons, where it is closely associated with lysosomal dysfunction [64]. GPNMB expression is elevated in aged liver and kidney tissues and correlates positively correlating with senescence‐associated β‐galactosidase (SA‐β‐Gal) activity [64]. Critically, GPNMB mediates phagocytic dysfunction in microglia, thereby exacerbating amyloid‐β (Aβ) deposition [69]. Thus, GPNMB serves as a valuable marker for assessing organ fibrosis and lysosome‐related senescence. In oncological contexts, CD97 is persistently overexpressed in glioma stem cells (GSCs), showing positive correlations with stemness‐associated genes SOX2 and OCT4; silencing CD97 reduces tumorigenic capacity by approximately 70% [64, 70]. CD97‐positive GSCs exhibit resistance to chemotherapy, and their targeted elimination prolongs survival in murine models by 40%, underscoring CD97's relevance in the study of senescence within tumor stem cells and the tumor immune microenvironment [70]. Dipeptidyl peptidase‐4 (DPP4), a cell surface glycoprotein responsible for the degradation of peptide substrates such as growth factors and cytokines, is frequently expressed in senescent cells (SnCs) [71]. Inhibition of DPP4 has been shown to reduce the production of matrix metalloproteinases (MMPs), thereby delaying tissue degradation and remodeling processes at sites of aging and cellular senescence. Additionally, DPP4 inhibition decreases macrophage accumulation and infiltration within inflamed vascular tissues [72]. Furthermore, selective targeting of DPP4‐positive senescent chondrocytes has been demonstrated to mitigate the progression of osteoarthritis [73]. Concerning matrix‐associated proteins, fibronectin secreted by senescent cells, facilitates macrophage‐mediated phagocytosis, while fibroblast activation protein (FAP), which is specifically expressed in senescent fibroblasts, has been targeted using CAR‐T therapy to alleviate fibrotic diseases [74]. Recent advances have identified platelet‐derived growth factor receptor β (PDGFRβ) as a significant extracellular matrix (ECM) marker in kidney fibrosis. Targeting PDGFRβ through in vivo CAR‐T therapy has been shown to ameliorate fibrosis‐associated pathologies across multiple chronic kidney disease models [75].

### 3.2 Cell Sources

Contemporary research on immune cell therapies predominantly emphasizes therapeutic efficacy, often overlooking critical considerations related to the cellular source. This emphasis is attributable to two principal factors. First, the majority of patients undergoing immune cell therapies present with advanced‐stage malignancies characterized by refractory disease and resistance to standard treatments, frequently rendering them unable to tolerate multiple treatment cycles. Second, although universal (“off‐the‐shelf”) cell therapies offer considerable potential due to their standardized manufacturing processes and immediate availability, current clinical evidence indicates limitations in their therapeutic effectiveness. Moreover, all presently approved cell therapy products are based exclusively on autologous immune cells. In contrast to malignant tumors, natural aging and age‐associated diseases represent chronic conditions with distinct pathophysiological features and therapeutic demands. Consequently, these conditions necessitate thorough investigations into diverse cellular sources to inform the development of optimized therapeutic strategies. (see Table 2)

**TABLE 2:** Manufacturing sources of engineered immune cells.

Cell types	Cell source	Proliferati‐on capacity	Biological authenticity	Clinical maturity	Key Considerations/ Limitations in Anti‐aging
Cell lines	RAW264.7/ THP‐1/ NK‐92	Unlimited	Low	Low	Not suitable for anti‐aging therapy research due to low biological authenticity and immortalizing mutations. Their use is largely confined to early‐stage mechanistic screening
Primary Immune‐Cells	PBMC/ BMDM/ Spleen	Limited	high	High	Donor age effect is a critical factor. Cells from aged donors may already be dysfunctional. For applications like immune rejuvenation, autologous sources may not be optimal.
HSCs	Bone marrow/ Cord blood/ peripheral blood	Moderate	Moderate (Blood specific)	High	A core candidate for reversing immunosenescence. Key challenges include obtaining fully functional HSCs from aged individuals and ensuring their successful engraftment and regeneration within the aged bone marrow niche.
IPSCs	Somatic cell	Unlimited	High	moderate	The most promising yet complex source. Advantages include access to "rejuvenated" autologous cells and the potential to differentiate into any desired cell type. Major hurdles encompass safety risks (tumorigenicity, genomic stability), differentiation efficiency, high cost, and lengthy preparation time

#### 3.2.1 Cell Lines

Cell lines are widely employed in in vitro studies due to their accessibility, unlimited proliferative capacity, and ease of exogenous genetic modification. For instance, the NK‐92 cell line, recognizes stress ligands (e.g., MICA/B, ULBP) expressed on senescent cells via NKG2D, thereby initiating cytotoxic responses [81]. Co‐culture experiments demonstrate that NK‐92 cells exhibit significant cytotoxic activity against radiation‐ or chemically induced senescent cells, with killing efficiency positively correlated with NK cell activation [82]. Notably, blocking the NKG2D receptor markedly diminishes this cytotoxic capacity [83]. To improve therapeutic efficacy, a team developed a chimeric polypeptide, E16‐uPA24, which simultaneously binds uPAR on senescent cells and mGluR5 on NK‐92 membranes [28]. This dual‐targeting approach enhanced NK‐92‐mediated clearance of senescent hepatocytes by two‐ to three‐fold in in vitro models, accompanied by elevated IFN‐γ secretion.

The murine macrophage‐like cell line RAW264.7 and human leukemia monocytic cell line THP‐1 have been utilized to generate CAR‐Ms for evaluating anti‐tumor potential [84, 85, 86, 87]. Despite the promising therapeutic prospects, It is crucial to distinguish between research tools and clinical assets. Immortalized cell lines like NK‐92, often derived from malignant origins, are invaluable for preclinical proof‐of‐concept studies. However, their tumorigenic potential generally precludes direct clinical application. The transition to human therapy necessitates manufacturing with clinical‐grade primary cells—such as those derived from peripheral blood, cord blood, or induced pluripotent stem cells (iPSCs)—under Good Manufacturing Practice (GMP) standards. This shift introduces immense challenges related to scalable expansion, stringent quality control, batch‐to‐batch consistency, and ultimately, prohibitive costs, forming a significant barrier to commercialization.

#### 3.2.2 Primary Immune Cells

Primary cells, derived directly from tissues such as peripheral blood, serve as the cornerstone for clinically translatable cellular therapies due to their intrinsic physiological function and lack of tumorigenic risk. Their application in anti‐aging strategies is particularly compelling, as they can be engineered to directly target the cellular and molecular hallmarks of aging. However, it is critical to note that the functional quality of primary immune cells is intrinsically compromised in aged donors, a phenomenon known as immunosenescence. This presents both a challenge (requiring rejuvenation strategies for autologous use) and an opportunity (allogeneic cells from young donors may offer superior function).

Engineered primary immune cells offer a dynamic approach for senescent cell clearance. Peripheral blood‐derived NK cells can naturally recognize and eliminate senescent cells via receptors like NKG2D [37]. To enhance this intrinsic surveillance, NKG2DL‐directed CAR‐T cells have been developed. In preclinical models of accelerated and natural aging, a single infusion of these CAR‐T cells significantly reduced senescent cell burden across multiple tissues, improved metabolic and physical function, and extended health‐span, demonstrating a direct impact on systemic aging [27]. In addition, primary cell‐based therapies show remarkable precision in reversing age‐related tissue fibrosis and chronic inflammation [25, 26]. Besides, a new version of CAR‐M, which recognizes the major inflammatory TNFα and activates an intracellular IL‐4 signaling pathway, thereby programming engineered macrophages for an anti‐inflammatory function with the help of synthetic biology. This kind of CAR‐M therapy demonstrated efficacy in multiple inflammatory disease models. Besides, aging is associated with a rise in autoimmunity, partly due to the breakdown of immune tolerance. CD19 CAR‐T cells, derived from patient peripheral blood, have induced profound and sustained drug‐free remission in patients with refractory autoimmune diseases like lupus [88, 89]. This strategy exemplifies a radical “immune system reset,” effectively depleting hyperactive autoreactive B‐cell clones. For aged individuals with concurrent autoimmunity, such an approach could not only treat the specific disease but also potentially reduce the chronic inflammatory burden that accelerates tissue aging.

Despite these promising outcomes, persistent hurdles impede clinical translation. The first is cell source limitations, obtaining sufficient numbers of functional primary cells from aged donors is challenging due to immunosenescence. Allogeneic sources from young donors are an alternative but introduce risks of graft‐versus‐host disease (GvHD) and rejection. Second is hostile aged microenvironment, the pro‐inflammatory, nutrient‐poor, and immunosuppressive milieu of aged tissues can impair the engraftment, persistence, and efficacy of infused therapeutic cells. Finally, the exorbitant cost and complex logistics of manufacturing autologous cell products are magnified for a large elderly patient population, raising critical accessibility issues.

#### 3.2.3 HSCs

Hematopoietic stem cells (HSCs) can be differentiated ex vivo into diverse immune effector subsets — including T cells, B cells, and natural killer (NK) cells — providing a stable and scalable source for cell therapies. These progeny can be further genetically engineered, for instance, to express chimeric antigen receptors (CARs). This is particularly promising for generating allogeneic “off‐the‐shelf” products with enhanced anti‐senescence or tissue‐reparative functions. For example, cord blood (CB)‐derived HSCs can be differentiated into NK cells (e.g., RNK001), which have demonstrated potent and sustained activity in preclinical models [90]. These CB‐HSC‐derived cells exhibit low immunogenicity due to minimal HLA‐II expression, making them attractive for universal application [91]. From an aging perspective, infusing such young, functionally robust immune cells could directly counteract the decline in immune surveillance (e.g., against senescent cells) and improve tissue homeostasis in aged recipients.

The widespread establishment of cord blood banks provides direct access to cryopreserved, “juvenile” HSCs that are inherently free from age‐related accumulative damage. This positions cord blood‐derived hematopoietic stem/progenitor cells (HSPCs) as a strategically valuable source for anti‐aging cellular therapeutics, aiming to reconstitute a more youthful and balanced immune system.

Translating this potential requires overcoming challenges in cost‐effective mass production and ensuring the safety of engineered cells (e.g., minimizing off‐target effects) [92]. More importantly, the therapeutic paradigm is evolving toward integrated strategies. A promising approach combines the clearance of endogenous, aged HSCs (e.g., via antibodies targeting senescence‐associated markers like CD150/CD47 [47]) followed by the transplantation of young, healthy HSPCs or their engineered derivatives. This “reset” of the hematopoietic system aims to durably re‐establish a more balanced lympho‐myeloid output, reduce systemic inflammation, and restore effective immune surveillance and tissue repair — addressing the root cause of immune‐driven aging.

#### 3.2.4 IPSCs

Compared to hematopoietic stem cells, iPSCs offer several advantages for anti‐aging cellular therapies, including wide availability, high expandability, great potential for personalized treatment, ease of genetic modification, and suitability as a universal donor for “off‐the‐shelf” products. This technology directly addresses a core limitation of aging: the decline in the number and function of an individual's native immune cells. By creating immune effectors from a genetically “young” pluripotent state, iPSC‐derived products can be engineered for enhanced senescence surveillance, tissue repair, and modulation of the aged inflammatory microenvironment.

Engineered iPSC‐derived NK (iNK) and CAR‐iNK cells are particularly promising for senolysis. Their homogeneous, potent, and scalable nature overcomes the functional variability of NK cells isolated from aged donors. Beyond cytotoxic clearance, iPSC‐derived MSCs (iMSCs) can be harnessed as a consistent source of secreted rejuvenative factors. These iMSCs can be engineered to optimize their secretome — rich in anti‐inflammatory cytokines, pro‐angiogenic factors, and regenerative signals — to actively suppress chronic inflammation, promote stem cell niches, and support tissue repair in aged organs, such as the heart or joints. Besides, The platform's flexibility allows for the development of disease‐specific therapies for age‐related conditions. For example, iPSC‐derived microglia or macrophages can be engineered for enhanced phagocytic clearance of amyloid‐beta plaques in Alzheimer's disease models. Similarly, iPSC‐derived CAR‐T cells targeting FAP could be deployed against the pathological fibrosis that underpins cardiac, pulmonary, and hepatic aging.

In addition, the genetic tractability of iPSCs allows for the creation of immune cells with functions tailored to overcome the challenges of the aged body. Aging tissues exhibit altered chemokine landscapes, iPSCs can be engineered to overexpress homing receptors (e.g., CXCR4 to improve bone marrow engraftment [93]) to ensure therapeutic cells reach their target niches. Also, the aged and inflamed microenvironment is rich in immunosuppressive signals, iPSC‐derived cells can be armored by knocking out inhibitory receptors (e.g., PD‐1 [94]) or introducing cytokine switches (e.g., IL‐15^+/+^/TGFβ^−/^‐ [95]) to maintain their activity. In addition, through gene editing (e.g., CRISPR‐mediated knockout of HLA class I/II molecules [96]), hypoimmunogenic iPSC lines can be created. This enables the mass production of universal cell products that evade immune rejection, a critical feature for widespread application in the elderly population who may be immunocompromised.

Collectively, these advances highlight iPSC technology's versatility in generating engineered immune cells with tailored therapeutic functions. However, critical barriers impede clinical translation: (1) Teratoma risk from residual pluripotency necessitates enhanced differentiation efficiency and functional stability [97] in aged niches; (2) Immune rejection and microenvironmental mismatch require engineered immune evasion [98, 99] — particularly vital in non‐lymphodepleted contexts like anti‐aging therapy.

### 3.3 Gene Delivery Technologies

In immune cell therapy, gene delivery system serve as the core tools for introducing exogenous genetic material into immune cells. The design of these methods directly impacts gene editing efficiency, the functional activity of the cells and overall clinical safety. Based on their technical characteristics, delivery systems can be categorized into two major classes: viral and non‐viral delivery systems. (see Table 3).

**TABLE 3:** Gene Delivery Technologies.

Vector types	Delivery system	Delivery Efficiency	Safety	Key Considerations/ Limitations in Anti‐aging
Viral vector‐based	RV	High	insertional mutagenesis	Enables long‐term, stable expression of therapeutic genes but carries a high risk of insertional tumorigenesis; suitable primarily for ex vivo modification and difficult to apply in systemic administration.
	LV	High	insertional mutagenesis	
	AAV	Middle	Hepatotoxicity	Limited cargo capacity (<4.7 kb); may induce hepatotoxicity and immune responses. A high prevalence of pre‐existing neutralizing antibodies in the elderly population can reduce efficacy or preclude repeated dosing
	AdV	High	High immunogenicity	systemic administration may trigger severe systemic inflammatory responses (e.g., cytokine storm), posing higher risks in aged individuals. Expression is transient due to immune clearance.
Non‐viral	LNP	Middle	Low cytotoxicity	Primarily targets the liver with poor systemic tropism; long‐term safety data remain limited; efficiency in delivering large DNA molecules is lower than for RNA.
	Electroporation	High	High cytotoxicity	Causes tissue damage and inflammation, and is mainly suitable for local or ex vivo applications.
	VLP	Middle	Middle cytotoxicity	Assembly and loading are complex, presenting challenges for large‐scale production; cargo capacity is limited.
	Transposon	Low	Middle cytotoxicity	Allows long‐term, stable expression of therapeutic genes, but integration efficiency is relatively low and carries a risk of low‐frequency random integration; immunogenicity is low

#### 3.3.1 Viral Vector‐Based Delivery System

In viral vector systems, retroviral vectors (RV), lentiviral vectors (LV), adeno‐associated viral vectors (AAV), and adenoviral vectors (AdV) represent the most widely utilized vector types. Globally approved CAR‐T products primarily rely on RV or LV vectors to deliver the gene sequence encoding the CAR into the T‐cell genome. This transduction endows T cells with the ability to specifically recognize and eliminate tumor cells. The core advantage of both RV and LV lies in their stable genomic integration capability, enabling long‐term expression of the CAR gene. However, RV can only transduce dividing cells, and its integration sites exhibit a propensity for regions near transcriptional start sites, posing a higher risk of insertional mutagenesis and consequently presenting safety limitations [100]. In contrast, LV possesses a broader cellular tropism, enabling efficient infection of both dividing and non‐dividing cells (such as primary T cells and stem cells). Nevertheless, its high production costs and the risk of random genomic integration remain significant challenges. To enhance safety, the self‐inactivating (SIN) design significantly reduces the probability of insertional mutagenesis by deleting the U3 region within the 3′ long terminal repeat (LTR), thereby eliminating enhancer/promoter activity [101]. Concurrently, process innovations are advancing; for example, WuXi Advanced Therapies' LentiVV system integrates packaging elements into the producer cell genome, requiring only a single transfer plasmid. This innovation substantially streamlines the manufacturing process and reduces costs.

AAV vectors are non‐enveloped, single‐stranded DNA viruses characterized by unique advantages of low pathogenicity and low immunogenicity [102]. The Nawaz team developed AAV‐delivered CAR Gene Therapy (ACG) technology. This approach involves intravenous injection of AAV vectors (such as AAV6) carrying the CAR gene, enabling the direct in vivo generation of CAR‐T cells within the host [103]. AAV vectors demonstrate distinct advantages in NK cell engineering. Studies indicate that capsid‐optimized AAV6 vectors can achieve transduction efficiencies of 60%–70% in NK cells [104], surpassing that of lentiviral vectors (40‐50%). Engineered NK cells produced using AAV vectors exhibited potent tumor‐killing activity in mouse models, with no observed significant graft‐versus‐host disease or aberrant genomic integration events. Furthermore, the AAV‐Cpf1 KIKO system, developed by Dai et al., enables the efficient one‐step generation of dual knock‐in CAR‐NK cells [105]. These cells displayed lower expression of exhaustion markers in CD22‐targeted therapy compared to conventional CAR‐NK cells.

AdV are characterized by high transduction efficiency and a large packaging capacity (up to 36 kb). However, their pronounced immunogenicity limits the feasibility of repeated administration [106]. The convergence of viral and non‐viral technologies has spurred the development of next‐generation delivery systems. For instance, adenovirus‐transposon hybrid vectors combine the efficient delivery capability of adenovirus with the stable genomic integration conferred by the transposase system [107, 108]. Alternatively, lipid nanoparticle (LNP)‐encapsulated viral vectors potentially offer the dual advantages of targeted delivery and reduced immunogenicity [109].

#### 3.3.2 Non‐Viral Delivery Systems

##### 3.3.2.1 Lipid Nanoparticles(LNP)

The successful deployment of LNP‐mRNA COVID19 vaccines has catalyzed a paradigm shift, demonstrating the feasibility of using non‐viral vectors for precise, in vivo genetic reprogramming. This technology is now rapidly converging with the field of cell therapy, offering a revolutionary alternative to traditional *ex vivo* cell engineering. The core advantages are profound: it bypasses the complex, costly, and time‐consuming processes of cell isolation, culture, genetic modification, and reinfusion, potentially enabling scalable and accessible “single‐shot” cellular therapies.

Recent breakthroughs have focused on overcoming the natural hepatic tropism of conventional LNPs to achieve targeted delivery to specific immune cell subsets. T cell‐targeted LNPs are developed through surface conjugation with CD8 antibodies or T‐cell‐specific ligands [110, 111]. This strategy achieved much elevated in vivo gene editing efficiency in target cells while significantly reducing off‐target accumulation in the liver, thereby improving the therapeutic window. For anti‐aging applications, this technology could be harnessed to directly edit endogenous, age‐compromised T cells or NK cells in situ, enhancing their senescence surveillance or reinvigorating their function without the need for cell extraction. To address inherent limitations of conventional LNPs, such as limited cargo capacity, immunogenicity, and batch‐to‐batch variability, innovative platforms are emerging. Engineered Endogenous Protein Assemblies (EASY) system from Westlake University utilized engineered protein condensates (ProteanFect), self‐assembles with nucleic acids into nanoparticles. It boasts an ultra‐high loading capacity (up to 50‐fold greater than LNPs) and can deliver diverse cargoes, including plasmid DNA, mRNA, and CRISPR‐Cas9 ribonucleoprotein (RNP) complexes. It's demonstrated 67%–88% editing efficiency in primary human NK and B cells ex vivo highlights its potency for engineering hard‐to‐transfect immune cells. Another hybrid vectors called Proteo‐Lipid Vehicles (PLVs) incorporate the FAST protein from fusogenic reovirus into lipid bilayers [112]. This design enables efficient cellular entry with low immunogenicity, allowing for repeated systemic administration—a critical requirement for chronic age‐related conditions. PLVs have successfully delivered genetic payloads to extrahepatic tissues like the lungs in preclinical models, expanding the scope for systemic in vivo cell therapy.

These advancements collectively represent significant progress in addressing the key challenges associated with traditional LNPs – namely, hepatic tropism limitations, immunogenicity risks, difficulties in delivering large genetic payloads, and complexities in manufacturing and storage stability. However, LNPs themselves can trigger innate immune responses, managing these reactions is particularly crucial for the aging population, which often experiences heightened inflammaging and multiple comorbidities. Future advancement of LNP and novel delivery platforms is steering toward sophisticated “in vivo cell engineering.” Key directions involve multi‐modal targeting strategies that combine passive (e.g., size, charge) and active mechanisms (e.g., multiple ligands) to achieve exceptional specificity, along with logic‐gated delivery systems activated only by multiple aging‐specific signals (e.g., a senescence marker plus a tissue‐specific antigen) for enhanced safety. These will be supported by advanced lipid and biomaterial designs offering improved biocompatibility and endosomal escape. Furthermore, integrating these platforms with controlled epigenetic reprogramming, such as the delivery of rejuvenating factors, will enable transcriptional‐level restoration of aged immune cell function.

##### 3.3.2.2 Electroporation

**FIGURE 3:**
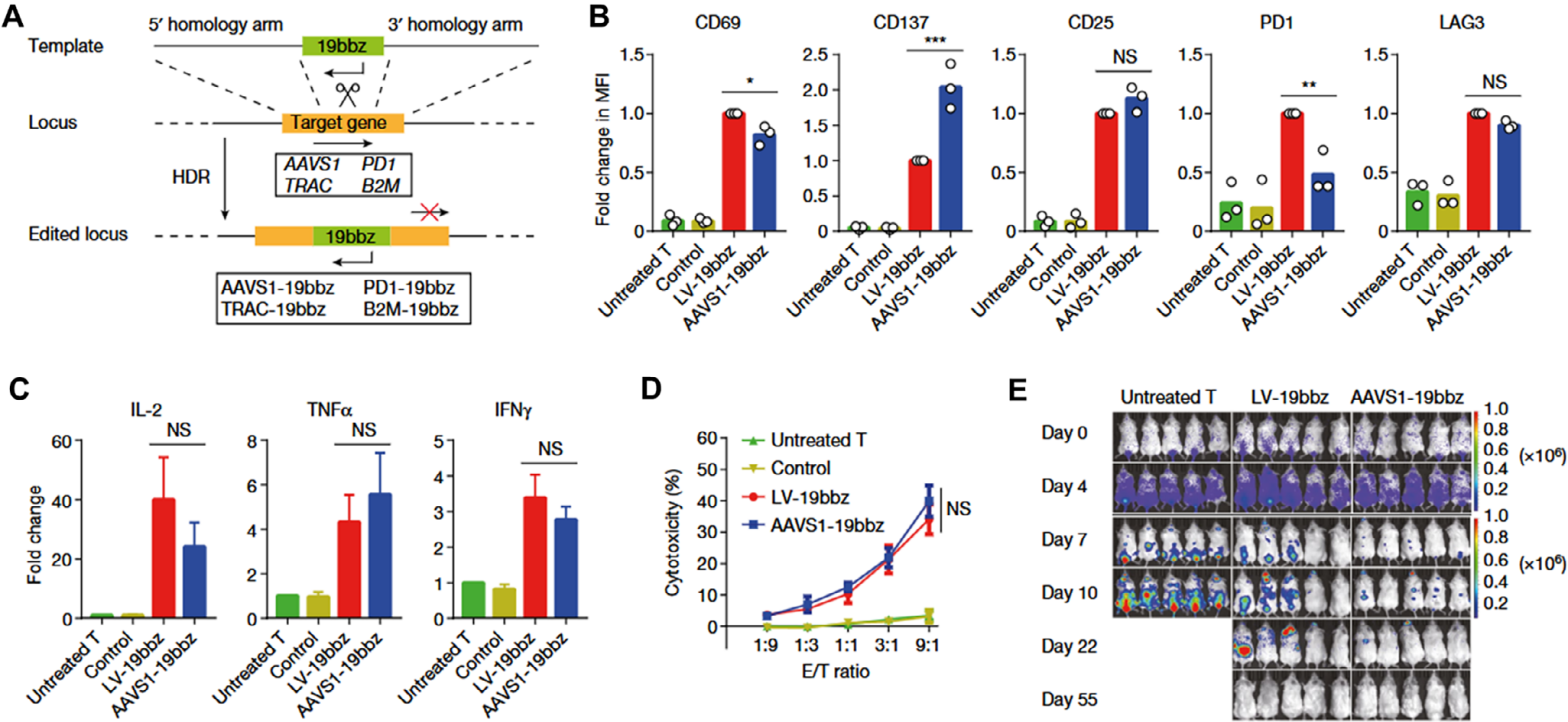
Comparable anti‐tumor efficacy was observed between CAR‐T cells generated through electroporation‐mediated integration into the AAVS1 locus and those produced via lentiviral transduction. (A) Specific integration of the CAR cassette into the target locus by homologous recombination through CRISRP‐Cas9. (B) MFI of CD69, CD137, CD25, PD‐1 and LAG3 expression in T cells after coculture with Raji cells for 24 h. (C) Cytokine secretion measured by bead‐based immunoassay in the supernatant after coculture with Raji cells for 24 h. (D) In vitro cytotoxicity against Raji cells as determined by lactate dehydrogenase (LDH) assay. (E) Bioluminescence imaging of tumor growth following different treatments on the indicated days after CAR‐T cell infusion. Reproduced with permission [114], Copyright 2022, Springer Nature.

Alternative approaches inducing enduring and transient CAR expression have also been investigated. Utilizing the PiggyBac transposon system combined with electroporation, mesoCAR‐T cells engineered via CAR gene delivery demonstrated potent cytotoxicity against the ovarian cancer cell line SKOV‐3, achieving 91–100% tumor elimination within 24 h [113]. In a preclinical study, Huang et al. established feasibility by using CRISPR/Cas9‐mediated electro‐transfection to precisely integrate an anti‐CD19 CAR sequence into the PD1 locus [114] (see Figure 3). This strategy concurrently induced PD1 interference and enhanced anti‐tumor activity.

Despite the advantages of electroporation in CAR cell production, its large‐scale clinical application faces significant challenges. Immune cells from diverse sources and subsets exhibit marked variations in sensitivity to electroporation parameters, necessitating individualized optimization protocols. For instance, optimal electroporation conditions for primary T cells and NK cells may differ by 2–3‐fold [115]. Furthermore, standardization remains underdeveloped across multiple stages—from circular plasmid preparation to electroporation parameter configuration. This gap is particularly pronounced for CRISPR RNP systems, where critical parameters such as the Cas9 protein‐to‐sgRNA ratio, complex assembly duration, and incubation temperature lack unified guidelines [116, 117]. Notably, the electroporation process itself may trigger cellular stress responses, leading to the release of pro‐inflammatory cytokines.

##### 3.3.2.3 Virus‐Like Particles (VLP)

**FIGURE 4:**
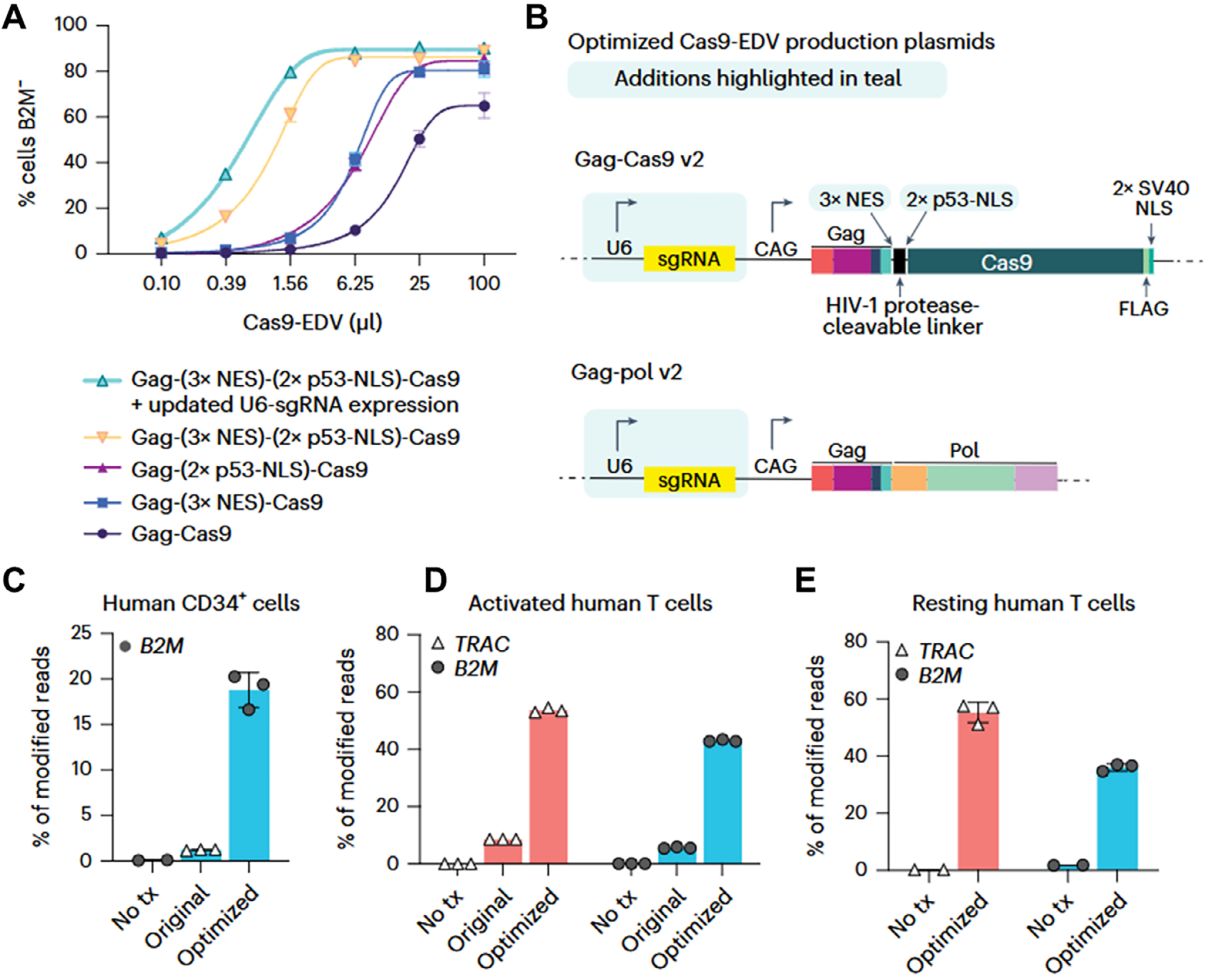
Optimization of cas9‐EDVs for enhanced genome editing activity in primary human cells. (A) Genome editing activity comparison of CD19 antibody targeted Cas9‐EDV variants packaging B2M‐targeted Cas9 RNPs. (B) Diagram of the optimized Gag‐Cas9 and Gag‐pol Cas9‐EDV production plasmids. (C‐E) Genome editing activity of optimized VSVG‐pseudotyped Cas9‐EDVs in primary human CD34+ cells (C), activated (D) and resting primary human T cells (E). Reproduced with permission [119], Copyright 2024, Springer Nature.

VLPs retain viral structural proteins but lack the viral genome, combining the advantages of high transduction efficiency and low pathogenicity. The dendritic cell‐targeted VLP (DVLP) platform, developed by Yin et al., enables the co‐delivery of mRNA and the display of antigenic proteins [118]. Through engineering with Sindbis virus glycoprotein (SV‐G) for dendritic cell‐specific targeting, DVLP significantly enhances anti‐virus immune responses [118] (see Figure 4). The Envelope Delivery Vehicle (EDV) platform achieves specific delivery of Cas9 ribonucleoprotein (RNP) to human T cells by engineering the display of scFV on a VSVGmut protein backbone [119]. This approach successfully generated genome‐edited CAR‐T cells in humanized mouse models. While VLPs exhibit considerable delivery potential, their in vivo delivery efficiency requires further improvement. Concurrently, the large‐scale production and purification of VLPs remain significant challenges. The development of efficient and cost‐effective manufacturing processes is essential to meet the demands of clinical translation.

##### 3.3.2.4 Transposon System

Limitations of traditional viral vectors for CAR‐T Cell production include prolonged manufacturing cycles (3‐6 weeks), high costs (over $400000 per treatment course), and the potential carcinogens risk due to insertional mutagenesis. By leveraging electroporation‐mediated one‐step gene integration, the transposon systems overcome these challenges and significantly enhance engineering efficiency and biocompatibility. Their potential safety advantages involve a more random integration profile, single‐copy integration capability, absence of viral components, and potential for excision.

In 2023, the MAJESTIC system achieved highly efficient and stable CAR gene integration in T cells [120]. This system combines an AAV vector (carrying the Sleeping Beauty transposon) with mRNA electroporation (encoding the SB transposase). Compared to lentiviral vectors alone, the MAJESTIC system demonstrated doubled CAR expression duration, 40% increase in cell viability, and exceeding 70% transduction efficiency. Besides, transposon technology improves the differentiation efficiency of iPSCs into immune cells through stable integration of differentiation‐regulating genes. For instance,. A team from Kyoto University employed the SB transposon to integrate the *FOXP3* gene into iPSC‐derived CD4^+^ T cells [121]. Induction with an AMRT cocktail (containing AS2863619, rapamycin, etc.) successfully generated immunosuppressive Treg‐like cells. These cells significantly prolonged survival in a xenogeneic GvHD mouse model.

On the one hand, Novel high‐activity transposons [122] (e.g., MAG) and integrated systems (e.g., MAJESTIC [120]) substantially improve the engineering efficiency and safety of CAR‐T/NK cells, simultaneously reducing manufacturing timelines. On the other hand, Clinical translation of transposon technology still faces hurdles, including control over integration sites, in vivo delivery efficiency, and scalable manufacturing processes.

### 3.4 Combination‐Enhanced Strategies

#### 3.4.1 Genetic Modification Enhancement

**FIGURE 5:**
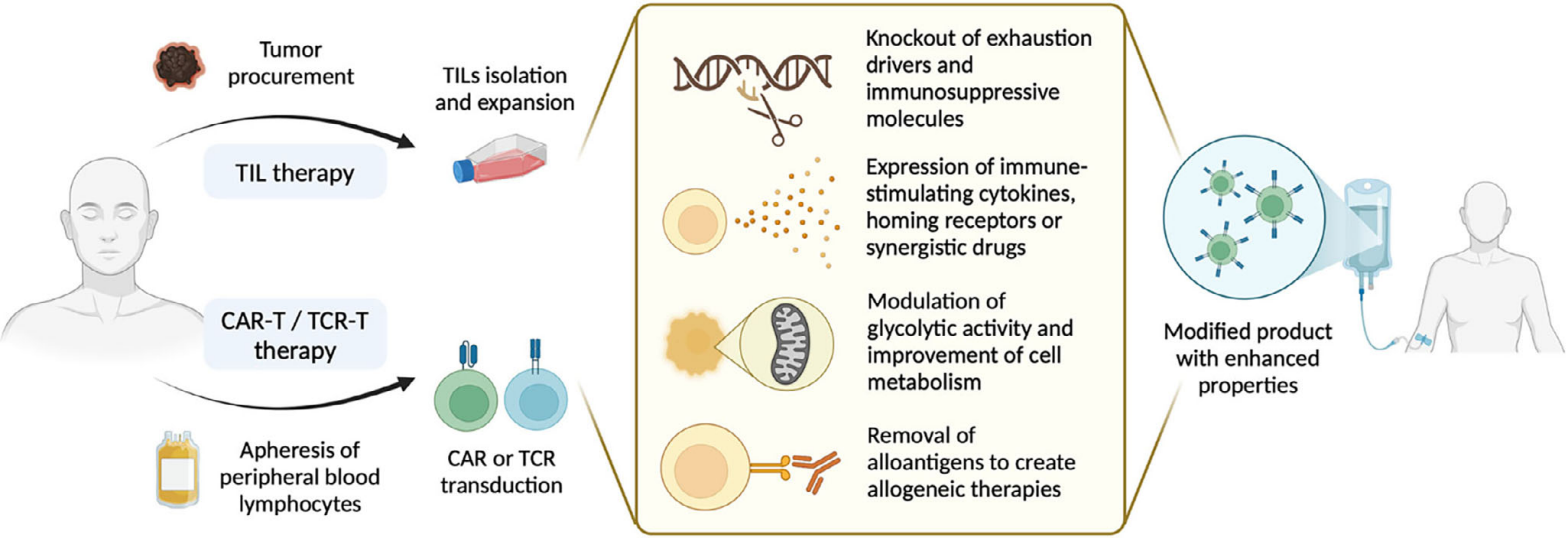
Overview of genetic engineering strategies and their applications in designing enhanced adoptive cell therapies. Reproduced with permission [124], Copyright 2025, Springer Nature.

The HIV‐specific synthetic Notch receptor (synNotch, CD4‐17b) developed by Yu's team enables precise functional control of CD8^+^ T cells. Upon recognizing viral antigen Env, this receptor induces controlled secretion of the broad‐spectrum neutralizing antibody VRC01 and bispecific T‐cell engagers (BiTE), achieving simultaneous neutralization of free viruses and killing of infected cells [123]. For cytokine engineering, CAR‐T cells enhance anti‐solid tumor activity via IL‐12/IL‐18 expression or improve persistence and memory phenotype through IL‐15 transduction [124]. Metabolic reprogramming strategies include boosting glycolysis (e.g., expressing glucose transporters or glycolytic enzymes like PFKFB3 to counteract metabolic suppression in the tumor microenvironment) and enhancing T‐cell activation signals by knocking out diacylglycerol kinase (DGK) [124]. To combat T‐cell exhaustion, CRISPR‐Cas9 silences exhaustion markers (e.g., PD‐1, LAG‐3, TOX—with careful preservation of memory function) or targets epigenetic regulators (e.g., suppressing transcription factor PRDM1) to reverse exhaustion [124]. Furthermore, CAR‐NK cells address poor in vivo persistence by expressing membrane‐bound IL‐15 and enhance antibody‐dependent cellular cytotoxicity (ADCC) via CD16 engineering [125], while CAR‐M overcomes immunosuppressive microenvironments by overexpressing pro‐inflammatory cytokines (e.g., IFN‐γ) and blocking phagocytosis‐inhibitory signals [124, 125]. (see Figure 5)

#### 3.4.2 Synergistic Effects of Multiple Immunotherapies

Strategies for synergistically overcoming solid tumor heterogeneity can be extended to senescent cell clearance, given the similarities in microenvironment and heterogeneity between senescent tissues and solid tumors. On one front, the combination of NK cells (rapid killing), macrophages (antigen presentation), or γδ T cells (deep tissue infiltration) offers a synergistic approach to tackle the heterogeneity challenge inherent in both solid tumors and senescent tissues. uPAR‐targeted CAR‐T cells effectively cleared senescent cells across multiple tissues (e.g., liver, lung) in aged mouse models [25], and reinfusion of ex vivo‐expanded autologous NK cells (aNK) reduced circulating senescence markers p16 and inflammatory cytokines IL‐6 in humans [37, 39], thereby combining aNK with CAR‐T may broaden tissue target coverage and enhance clearance efficiency.

On another front, addressing the key limitation of poor persistence and expansion of CAR‐T cells within solid tissues, BioNTech's researchers have developed innovative strategies. These include combining CLDN6‐targeted CAR‐T with an RNA vaccine encoding CLDN6 (CAR‐Vac), which promotes antigen‐presenting cells to express the target antigen, thereby providing continuous stimulation for CAR‐T cell expansion and activation. This combination demonstrated significant synergy in a Phase I/II clinical trial [126]. Another explored strategy combined a WT1 mRNA vaccine with a DC/AML fusion cell vaccine alongside CAR‐T therapy [127]. This approach leverages dendritic cells (DCs) for efficient presentation of leukemia stem cell‐associated antigens, enhancing CAR‐T recognition of senescent cells within the tumor microenvironment. Results showed that 44% of patients elicited WT1‐specific T‐cell responses, and overall survival was significantly prolonged compared to conventional chemotherapy [127].

#### 3.4.3 Combination of Cell Therapies and Immunomodulatory Drugs

Current monotherapies exhibit significant limitations. Cell therapies, such as CAR‐T or NK cell infusions, can effectively identify and clear senescent cells [25], but their sustained activity is constrained by the immunosuppressive microenvironment. In elderly patients, immune‐senescence leads to diminished autologous T‐cell function, limiting the efficacy of ex vivo expanded cell reinfusion. Small‐molecule immunomodulators like rapamycin can improve immune function but lack target specificity, require chronic administration, and carry risks of systemic adverse effects (e.g., rapamycin‐induced hyperglycemia and dyslipidemia [128]). Combining cellular immunotherapy with immunomodulatory drugs offers a promising strategy to overcome these barriers by concurrently enhancing immune cell function and ameliorating the microenvironment. For instance, a strategy combining uPAR‐targeted CAR‐T cells with rapamycin demonstrated sustained anti‐aging effects in animal models [25]. Further studies revealed rapamycin's dual immunomodulatory roles: it inhibits mTOR signaling to suppress SASP secretion while promoting CAR‐T cell differentiation toward a memory phenotype, extending their in vivo persistence. Metformin, activating the AMPK pathway, improves T‐cell mitochondrial function, boosting proliferation and effector capacity, while concurrently reducing insulin/IGF‐1 signaling [129] – a key regulator of senescence. In a human trial, autologous NK cell infusion combined with oral metformin and rapamycin significantly reduced circulating p16 and IL‐6 for 6‐12 months [130]. In patients with age‐related degenerative arthritis, this combination reduced joint pain scores by 50% and markedly improved mobility [131].

Given the heterogeneity of senescence, immune phenotype‐guided combination strategies should be prioritized. Allogeneic NK cell (aNK) infusion with chimeric peptides [28, 37] for patients exhibiting low NK activity and rapamycin and/or metformin combined with cell therapy for those with high inflammatory burden [130].

## 4 Clinical and Preclinical Trials of Cellular Immunotherapies for Targeting Aging and Aging‐Associated Diseases

### 4.1 Aging and Aging‐Related Pathologies

Cellular immunotherapies, notably CAR‐T cell therapy, have demonstrated significant efficacy in the treatment of malignancies, particularly hematologic cancers. Consequently, there is a growing body of research investigating their applicability to non‐oncological conditions, including aging [132, 133]. uPAR is widely and highly expressed in various models of cellular senescence, while exhibiting minimal expression in normal tissues, thereby representing a promising target for senescent cell clearance strategies. Capitalizing on this expression profile, researchers have engineered uPAR‐targeted CAR‐T cells and validated their potent capacity to eliminate senescent cells both in vitro and in vivo, achieving clearance rates exceeding 80%. In disease models, this therapeutic approach has demonstrated sustained efficacy, for example, in models of high‐fat diet‐induced liver fibrosis and insulin resistance, uPAR‐CAR‐T cells effectively and persistently ameliorated metabolic dysfunction [25]. Despite these encouraging preclinical findings, several limitations constrain the clinical translation of this strategy. First, uPAR expression is not exclusive to senescent cells but is also observed in various physiological processes such as wound healing, as well as pathological conditions including inflammation, fibrosis, and cancer. This overlap, referred to as “target duality,” raises concerns regarding potential off‐target effects on healthy cells and the disruption of normal physiological functions. Second, the uniformity of uPAR expression across senescent cells derived from different tissues and individuals remains uncertain, necessitating further validation in human subjects to establish its reliability as a universal marker of senescence and to delineate an effective therapeutic window and clearance threshold. Moreover, given that anti‐aging interventions may require administration over extended periods, potentially spanning decades, the ultra‐long‐term safety profile of such therapies remains unknown. Consequently, under current technological limitations, the application of uPAR‐CAR‐T therapy is more feasibly confined to specific senescence‐associated diseases characterized by elevated uPAR expression — such as liver and pulmonary fibrosis — rather than serving as a systemic, broad‐spectrum preventive anti‐aging modality. Future research directions include the development of dual‐targeting systems that combine uPAR with additional markers to enhance targeting specificity, as well as the integration of artificial intelligence with multi‐omics datasets to accurately identify patient subpopulations most likely to benefit from uPAR‐targeted interventions.

Similarly, NKG2D ligands are expressed at low levels in certain activated normal cells, including immune and epithelial cells, paralleling the expression pattern of uPAR and thereby posing potential off‐target risks for therapies directed against these molecules. Furthermore, the efficacy of such immunotherapeutic approaches is inherently challenged in elderly populations due to immunosenescence. For instance, NK cells exhibit functional decline and diminished surface expression of the NKG2D receptor with advancing age, substantially impairing the effectiveness of in vivo engineered therapies reliant on these immune components. Finally, the long‐term safety — such as the potential adverse effects arising from excessive elimination of normal tissues — and the durability of therapeutic efficacy following a single intervention require comprehensive validation through further preclinical and clinical investigations.

**FIGURE 6:**
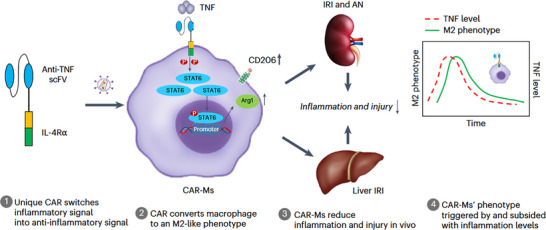
CAR‐Ms as inflammatory disease immunotherapy. Reproduced with permission [29], Copyright 2025, Springer Nature.

Considering that the upregulation of SASP intensifies the aging process, a novel CAR‐M platform is engineered to transform the pro‐inflammatory microenvironment into anti‐inflammatory signals, thereby promoting a stable M2 macrophage phenotype characterized by sustained anti‐inflammatory activity [29] (see Figure 6). In experimental models of acute kidney injury (AKI) and chronic kidney disease (CKD), treatment with CAR‐M significantly mitigated renal damage, decreased serum creatinine concentrations, reduced neutrophil infiltration, enhanced renal function, and attenuated fibrosis [29]. Likewise, in models of acute liver injury, administration of CAR‐M substantially diminished tissue necrosis, lowered alanine aminotransferase (ALT) and aspartate aminotransferase (AST) enzyme levels, and suppressed the production of inflammatory cytokines.

### 4.2 Neurodegenerative Diseases

The intricate bidirectional interactions between immuno‐senescence and neurodegenerative diseases such as Alzheimer's disease (AD) and Parkinson's disease (PD) reveal a pathogenic network where aged, dysfunctional immune cells directly drive neuroinflammation and pathology. This nexus presents not merely a disease mechanism but a suite of actionable therapeutic targets. Cellular immunotherapies are now being engineered to directly intervene at these critical junctures, offering a paradigm shift from passive observation to active immune system reprogramming within the central nervous system.

A core deficit in AD is the failure of brain‐resident microglia to clear pathological protein aggregates like β‐amyloid (Aβ), coupled with their shift toward a pro‐inflammatory, senescent state [134, 135]. CAR‐M are engineered to overcome recognition failure and correct this dual dysfunction. By expressing receptors targeting Aβ, these cells are directed to phagocytose plaques with high specificity [136]. This approach could be further enhanced by nano‐biomaterial ‐ assisted delivery; for instance, coupling CAR‐M cells with iron oxide nanoparticles allows for magnetic guidance to enrich therapeutic cells at disease sites, improving plaque clearance while concurrently reducing overall neuroinflammation. Beyond mere clearance, next‐generation strategies focus on reprogramming microglia via gene editing. Using CRISPR‐based tools or nanoparticle‐delivered modulators, endogenous microglia can be genetically instructed to overexpress anti‐inflammatory cytokines or Aβ‐degrading enzymes, thereby converting them from drivers of disease into agents of tissue homeostasis and repair.

The aged adaptive immune system contributes to neurodegeneration through both loss of protective functions and gain of cytotoxic ones. Immuno‐senescence leads to clonal expansions of cytotoxic CD8+ T cells that can infiltrate the brain and release harmful molecules [137]. Conversely, Treg function often declines with age [138, 139]. Precision T cell therapies are being developed to rebalance this axis. One innovative approach involves creating antigen‐specific ‐Tregs. In AD, Tregs engineered to express a TCR targeting Aβ can be selectively recruited to pathological sites [140]. Upon antigen recognition, these cells exert their potent local immunosuppressive functions, inhibiting pro‐inflammatory microglia and cytotoxic T cell activity, thereby dampening the overall neuroinflammatory milieu without broad systemic suppression. This strategy directly addresses the immune dysregulation while leveraging the brain's own pathology as a homing signal.

Neurodegeneration is fueled not only by local brain inflammation but also by systemic immuno‐senescence, which compromises the blood‐brain barrier (BBB) and allows inflammatory mediators to invade the CNS. Therefore, systemically administered cellular therapies that target aged immune compartments hold neuroprotective potential [141]. Approaches include adoptive transfer of rejuvenated immune cells, such as ex vivo‐expanded NK cells, to restore the declining surveillance against senescent cells throughout the body, including at the BBB and within the brain parenchyma [142]. This can reduce the peripheral burden of SASP factors that exacerbate neuroinflammation. Furthermore, emerging in vivo reprogramming strategies using targeted LNPs aim to genetically edit circulating T cells or hematopoietic stem cells to resist exhaustion or enhance anti‐inflammatory profiles [143], aiming to systemically rejuvenate the immune landscape and indirectly confer neuroprotection.

**FIGURE 7:**
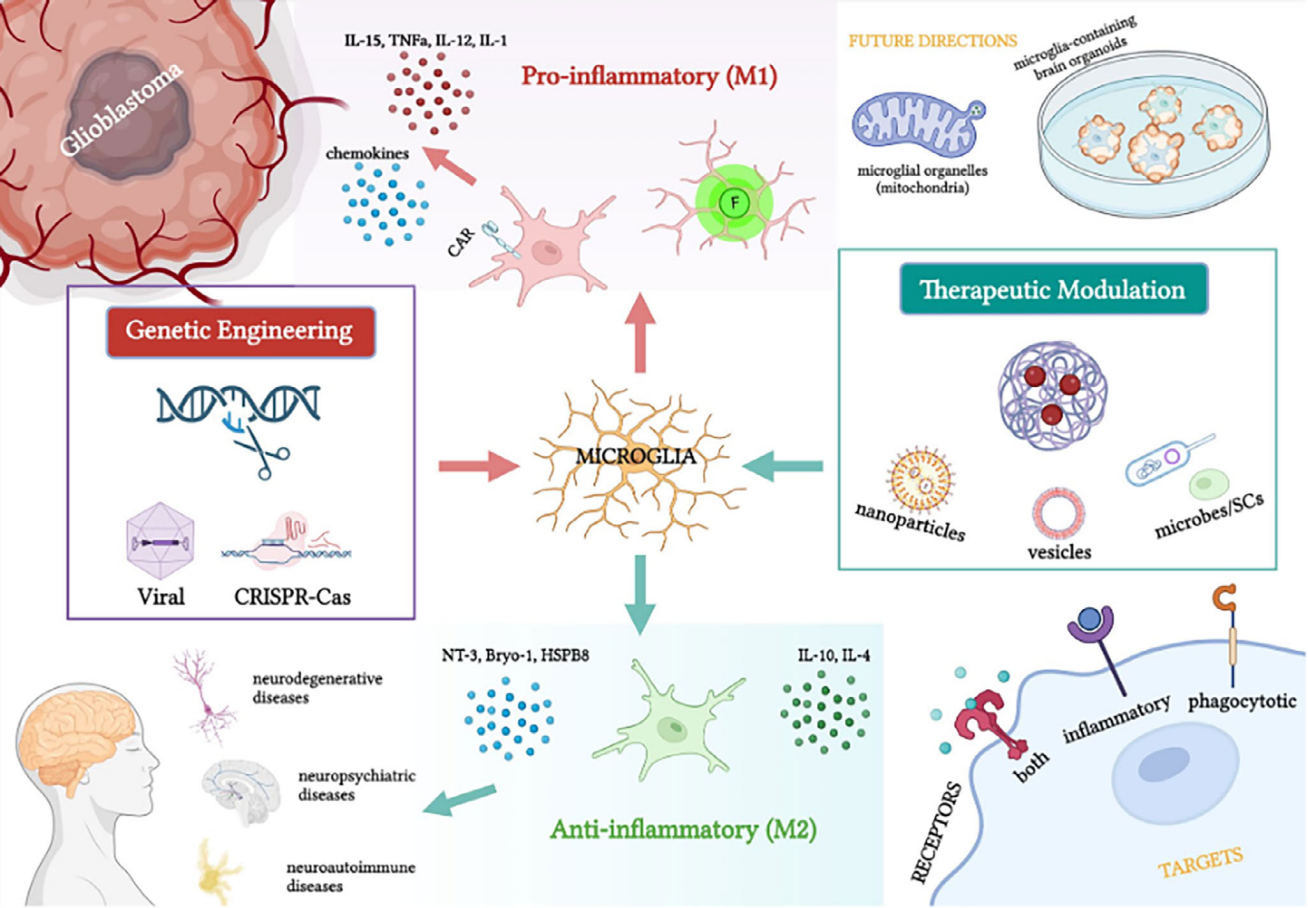
Overview of the engineered/reprogrammed microglia serving as therapeutic carriers and microenvironment modulators to treat a wide range of neurological disease. Reproduced with permission [144], Copyright 2024, Springer Nature.

The development of cellular immunotherapies for neurodegenerative diseases represents a logical and targeted response to the mechanistic understanding of immuno‐senescence. By moving beyond descriptions of pathology, these advanced strategies—including CAR‐M for targeted plaque clearance, CAR‐Tregs for precise immunomodulation, and systemic immune rejuvenation (see Figure 7) — are designed to directly intercept the specific immune dysfunction that drives neuronal damage. The convergence of cellular engineering, gene editing, and delivery technologies is thus creating a new frontier for treating neurodegeneration at its immunological roots, offering hope for interventions that are both precise and potent. However, the selection of therapeutic targets for neurodegenerative diseases presents a significant challenge due to the ubiquitous distribution of pathological proteins, such as Aβ and Tau, throughout the brain and their involvement in essential physiological functions. Furthermore, modulating neuroinflammation embodies a double‐edged sword. While excessive neuroinflammation is pathogenic, its complete or overzealous suppression may inadvertently impair essential protective immune responses. Consequently, therapeutic strategies must achieve a precise spatiotemporal modulation of the inflammatory milieu to maintain this delicate balance.

### 4.3 Aging‐Associated Autoimmune Diseases

Immuno‐senescence and autoimmune diseases exhibit a dynamic bidirectional interplay. On one hand, immuno‐senescence promotes the development of autoimmune disorders through multiple mechanisms: (1) The chronic inflammatory milieu of “inflammaging” facilitates aberrant self‐antigen release, activating autoreactive T and B cells, thereby triggering diseases such as systemic lupus erythematosus (SLE) and rheumatoid arthritis (RA); (2) Age‐related decline in immune surveillance impairs the clearance of self‐antigens, leading to the accumulation of abnormal cells and autoantibodies [145]; (3) Latent viral infections (e.g., cytomegalovirus, CMV) drive excessive memory T‐cell expansion, exacerbating immune dysregulation and autoimmune susceptibility. Conversely, autoimmune diseases reciprocally accelerate immune‐senescence: Chronic inflammation and sustained immune activation in patients induce T‐cell exhaustion and telomere shortening, resulting in premature immune aging. Furthermore, prolonged use of glucocorticoids or immunosuppressants may suppress thymic function and impair immune cell regeneration, creating a vicious cycle. This intricate crosstalk underscores their mutual reinforcement in disease pathogenesis, highlighting the need for targeted therapeutic strategies to disrupt this deleterious feedback loop (Table 4).

**TABLE 4:** Ongoing Clinical trials in autoimmune disease based on immune cell‐therapy.

Intervention/ Treatment	Disease/ Condition	Status	Phase	Study ID (https://clinicaltrials.gov/ accessed on 8 April 2025)	Location
CD19 USACAR‐T	Chronic or Refractory Primary Immune Thrombocytopenia	Recruiting	1/2	NCT06352281	China
CD19 CAR‐NK	systemic sclerosis, idiopathic inflammatory myopathies	Recruiting	1	NCT06733935	USA
tolDC‐VitD3	Multiple Sclerosis	Recruiting	1	NCT02903537	Spain
DSG3‐CAART /CD19CAR‐T	Pemphigus Vulgaris	Recruiting	1	NCT04422912	USA
MuSK‐CAART	Anti‐MuSK‐antibody‐positive Myasthenia Gravis	Recruiting	1	NCT05451212	USA
CD19 CAR‐NK	Systemic Lupus Erythematosus	Recruiting	1	NCT06518668	USA
CD19 CAR‐T	Idiopathic Inflammatory Myopathy	Recruiting	1/2	NCT06154252	USA/UK
BCMA‐CD19 cCAR‐T	Refractory Immune Thrombocytopenia	Recruiting	1	NCT06787989	China
NK+ Ocrelizumab	Refractory Primary and Secondary Progressive Multiple Sclerosis	Recruiting	1	NCT06677710	USA
CD19 CAR‐T	Refractory Progressive Multiple Sclerosis	Active, not recruiting	1	NCT06451159	USA

#### 4.3.1 Systemic Lupus Erythematosus (SLE)

SLE is a chronic autoimmune disease characterized by multi‐system involvement and the production of autoantibodies. Traditional therapies relying on glucocorticoids and immunosuppressants are associated with severe side effects and fail to achieve long‐term remission. In recent years, cellular immunotherapy has emerged as a research hotspot in SLE treatment due to its precision targeting and potential for durable disease control. CAR‐T therapy, which involves genetically engineering T cells to specifically target the B‐cell surface antigen CD19, eliminates aberrantly activated B cells and plasma cells, thereby reducing autoantibody production. A landmark study reported that 15 patients with autoimmune diseases (including 8 SLE cases) achieved sustained drug‐free remission after CD19 CAR‐T treatment [88]. Another team demonstrated the safety and efficacy of this approach in 20 pediatric refractory SLE patients: 15 patients achieved a SLE Disease Activity Index‐2000 (SLEDAI‐2K) score below 4 within three months, accompanied by a significant reduction in glucocorticoid dosage [89]. Moreover, CD19 CAR‐T therapy not only depletes pathogenic B cells but also restores immune tolerance by modulating T‐cell subsets, such as increasing the proportion of Tregs [89]. However, in a subset of patients, the disease may be driven by long‐lived plasma cells expressing B‐cell maturation antigen (BCMA) but lacking CD19 expression. Consequently, even after receiving CD19‐targeted CAR‐T cell therapy, autoantibodies persist in the body due to incomplete eradication of these BCMA and CD19 negative pathogenic plasma cells. Teclistamab is a BCMA‐ and CD3‐targeting off‐the‐shelf bispecific T‐cell engager that binds simultaneously to T cells and B cells, thereby activating T cells to eradicate pathogenic B cells. It has been approved for the treatment of multiple myeloma. Following subcutaneous administration of teclistamab, SLE patients exhibited a significant reduction in double‐stranded DNA antibody levels, normalization of complement levels and type I interferon activity, along with substantial depletion of peripheral B cells and bone marrow plasma cells by week 8 of treatment [146]. Clinical studies demonstrated favorable safety profiles of teclistamab, with no observed neurotoxicity or myelotoxicity, and only low‐grade cytokine release syndrome being reported.

These findings highlight the dual therapeutic potential of CAR‐T or endogenous T cells in both eradicating autoreactive cells and rebalancing immune homeostasis, offering a transformative strategy for SLE management.

#### 4.3.2 Rheumatoid Arthritis (RA)

**FIGURE 8:**
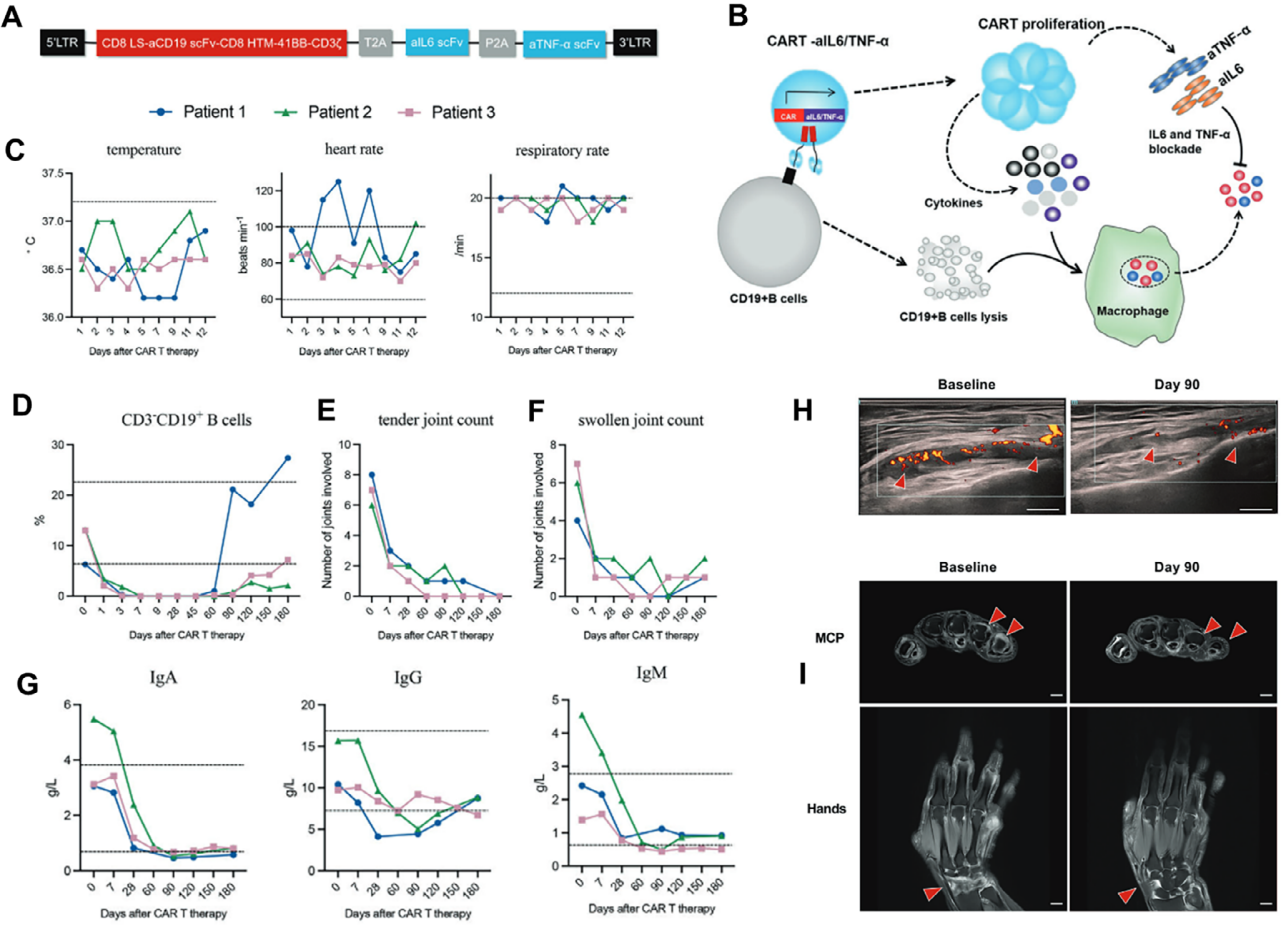
Clinical safety and efficacy of CD19/aIL‐6/aTNF α CAR‐T cells in RA. (A) Design of CAR construct and sequence of anti‐IL‐6 scFV and anti‐TNF α. (B) Schematic illustration of CAR‐T cells depleting CD19+ B cells and secreting scFV of immunoglobulins against IL‐6 and TNF α. (C) Body temperature, heart rate and respiratory rate taken at the same time of the day after treatment with CAR T‐cells. (D) Circulating CD19+ B cells in the patients’ peripheral blood. (E‐F) Effects of CAR‐T cells on tender (E) and swollen (F) joint counts. (G) Effects of CAR T‐cell therapy on serum levels of immunoglobulins G (IgG), A (IgA), and M (IgM). (H) Representative images of ultrasound Power Doppler (PD) signal (arrowheads) in the knee joint before and 3 months after CAR T‐cell therapy in patient 2. (I) Representative MRI scans showing improved synovitis (arrowheads) of the hands and metacarpophalangeal joint (MCP) at baseline and 3 months after CAR T‐cell treatment in patient 2. Reproduced with permission [147], Copyright 2025, Springer Nature.

RA is a chronic autoimmune disease characterized by persistent synovitis and systemic immune dysregulation. Conventional therapeutic approaches exhibit limited efficacy in refractory cases. In a pilot clinical trial involving three refractory RA patients, a fourth‐generation CD19‐targeted CAR‐T therapy achieved rapid B‐cell depletion (peripheral B‐cell clearance within 3–7 days, followed by reconstitution by day 60), significantly reduced disease activity (as measured by the DAS28 score), and normalized levels of autoantibodies (rheumatoid factor and anti‐cyclic citrullinated peptide antibodies) [147] (see Figure 8). Notably, no severe adverse events, such as CRS were observed. The therapeutic mechanism involves targeted elimination of pathogenic B cells, thereby disrupting the self‐perpetuating cycle of autoantibody production, synovial fibroblast activation, and osteoclast differentiation in RA. Furthermore, engineered CAR‐T cells secrete neutralizing antibodies against IL‐6 and TNF‐α, enabling dual effects of metabolic reprogramming and local anti‐inflammatory action to enhance therapeutic outcomes. However, prolonged B‐cell depletion may increase infection risks, necessitating close monitoring of sustained hypogammaglobulinemia.

Previous studies have demonstrated that MSCs restore immune homeostasis by suppressing T/B‐cell activation, modulating macrophage polarization (from pro‐inflammatory M1 to anti‐inflammatory M2 phenotypes), and upregulating regulatory T‐cell proportions. Clinical trials of MSC transplantation reported rapid reductions in inflammatory cytokines (e.g., IL‐1β, IL‐6) and improved joint function, though partial relapse occurred in some patients [148], highlighting the need for long‐term efficacy validation. Future directions may explore combination therapy integrating CAR‐T cell therapy with MSC transplantation, which could synergistically combine immune clearance and tissue repair. This approach holds promise for addressing both pathogenic immune responses and structural damage in refractory RA, potentially achieving sustained remission.

#### 4.3.3 Myasthenia Gravis (MG)

MG is an autoimmune disorder mediated by acetylcholine receptor antibodies (AChR‐Ab), with its central mechanism involving immune‐mediated attack at the neuromuscular junction (NMJ), resulting in muscle weakness. In 2023, the first global case of anti‐CD19 CAR‐T cell therapy for refractory MG was reported. A female patient treated with autologous CD19‐targeted CAR‐T (KYV‐101) achieved profound peripheral B cell depletion, a 70% reduction in AChR antibody titers, and significant clinical improvement (e.g., restored independent ambulation), without cytokine release syndrome (CRS) or neurotoxicity. This therapy selectively eliminates CD19+B cells and plasmablasts, halting pathogenic autoantibody production while preserving long‐lived plasma cells in the bone marrow to maintain protective antibody levels [149]. Compared to conventional anti‐CD20 monoclonal antibodies (e.g., rituximab), CAR‐T cells demonstrate enhanced efficacy by penetrating the central nervous system (CNS) to eradicate compartmentalized B cells and exhibiting prolonged therapeutic durability.

Moreover, Descartes‐08, an mRNA‐based CAR‐T therapy targeting B cell maturation antigen (BCMA), is designed to deplete long‐lived plasma cells. In a phase 1b/2a clinical trial, three refractory MG patients showed a mean improvement exceeding 50% on the Myasthenia Gravis Composite (MGC) scale post‐treatment, with no severe adverse events reported [150]. By enabling transient CAR expression, this approach minimizes long‐term immunosuppressive risks, offering a novel strategy for precision immunotherapy in MG.

Current cellular immunotherapies face significant challenges primarily centered on target specificity, efficacy sustainability, and long‐term safety. The existing targets, such as CD19 and BCMA, are predominantly effective against the B‐cell lineage, offering limited efficacy for autoimmune diseases mediated by T cells or innate immunity, constraining therapeutic window and the range of applicable diseases. Furthermore, the potential for on‐target, off‐tissue toxicity, which poses a substantial risk of long‐term immunodeficiency and increased susceptibility to infections. The immunosuppressive and inflammatory microenvironment characteristic of many autoimmune diseases can hinder the expansion of administered therapeutic cells and promote their premature exhaustion, thereby limiting durable responses. Additionally, a major translational hurdle is the limited capacity of these cells to effectively traffic and infiltrate certain diseased tissues, such as the central nervous system. From a manufacturing perspective, developing universal, low‐immunogenicity allogeneic cell products remains technically challenging, which impacts scalability and cost‐effectiveness. Perhaps the most critical concern for clinical translation is the scarcity of long‐term safety data. The profound and potentially permanent reshaping of the immune system and the unknown effects on future autoimmune responses require rigorous, long‐term patient follow‐up to fully understand the risk‐benefit profile of these interventions.

### 4.4 Cardiovascular Diseases

The established link between immuno‐senescence and cardiovascular disease — centered on chronic inflammation, immune dysfunction, and metabolic dysregulation — provides a clear roadmap for therapeutic intervention. Instead of merely mitigating symptoms, next‐generation cellular immunotherapies are being engineered to directly target and disrupt these core pathogenic mechanisms, offering a paradigm shift toward causality‐based treatment.

**FIGURE 9:**
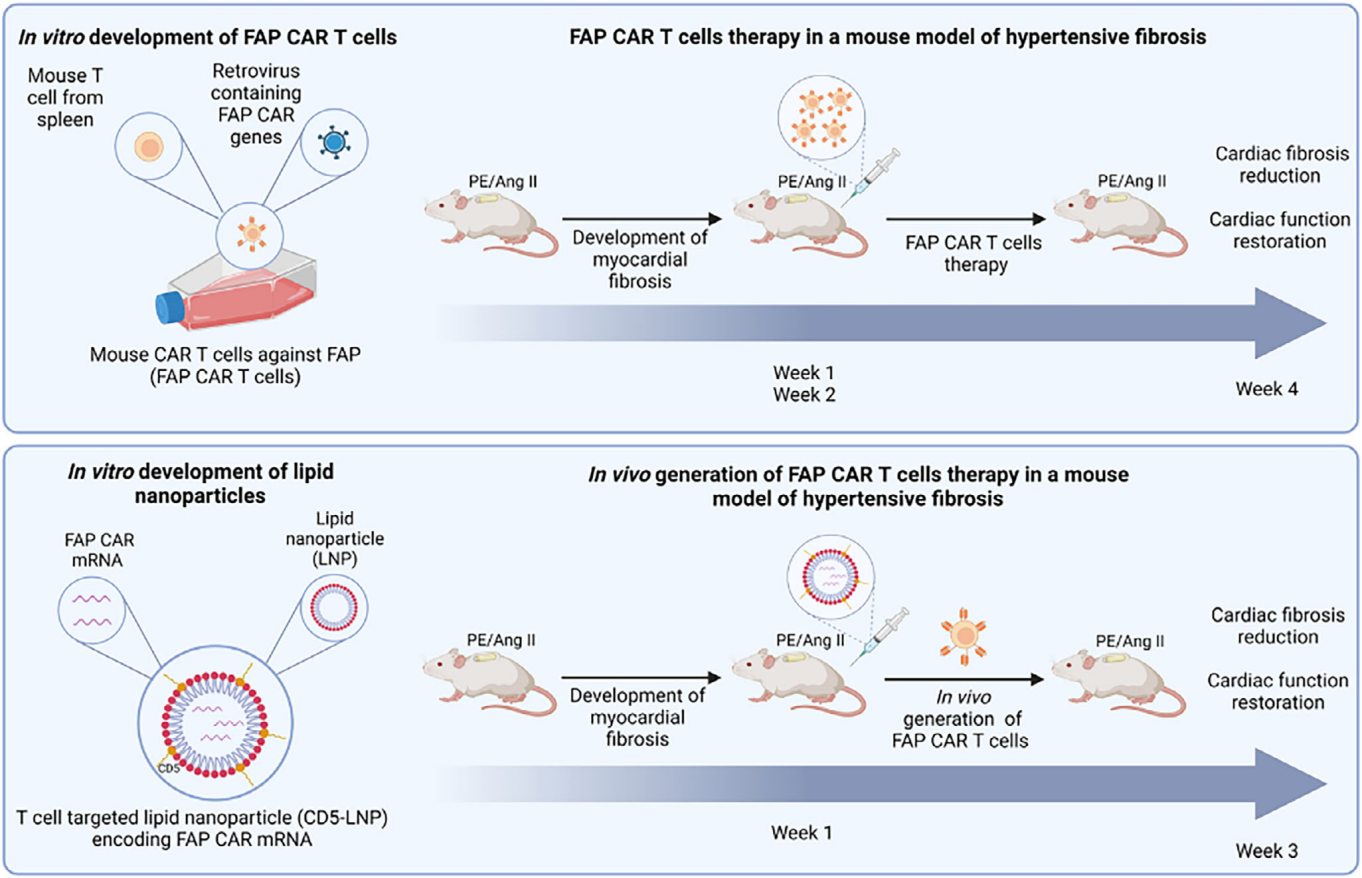
In vitro and in vivo FAP CAR‐T cell generation strategies for hypertensive fibrosis treatment in a mouse mode. Reproduced with permission [157], Copyright 2023, Elsevier Masson SAS.

Cardiac fibrosis, a final common pathway in heart failure driven by activated fibroblasts, represents a prime target. While conventional drugs offer limited efficacy, FAP‐directed CAR‐T cells are designed to seek out and eliminate FAP‐expressing activated fibroblasts in the heart [151] (see Figure 9). Preclinical studies in pressure‐overload models demonstrate that a single infusion of these cells can significantly reduce fibrosis, improve cardiac compliance, and restore function, with effects sustained over time. To address the complexity and cost of *ex vivo* cell manufacturing, mRNA ‐ LNP technology is being adapted for cardiovascular therapy. Preclinical studies show that intravenous infusion of mRNA‐LNPs encoding for FAP‐CAR can temporarily reprogram circulating T cells in situ, generating a transient population of CAR‐T cells within the host [152]. This “hit‐and‐run” approach has demonstrated efficacy in reducing fibrosis in mouse models and offers advantages in scalability, reduced manufacturing complexity, and potentially improved safety due to the transient nature of the modified cells. To further enhance safety profiles, alternative platforms like CAR‐NK or CAR‐M cells are under exploration [153, 154, 155]. These leverage the innate biology of NK cells and macrophages — such as shorter lifespans and natural tissue‐remodeling capacities — to potentially achieve effective fibrosis clearance with a lower risk of persistent cytotoxicity and cytokine release syndrome associated with conventional CAR‐T therapy.

Post‐infarct heart failure is propelled by maladaptive, non‐resolving inflammation. Cellular therapies aim to recalibrate this immune response. Treg therapy is a prominent strategy, wherein expanded autologous or allogeneic Tregs are administered to suppress excessive inflammation, promote transition to a reparative phase, and reduce adverse remodeling. Engineering these cells to express homing receptors (e.g., to target the infarcted myocardium) is an active area of research to increase local efficacy. Beyond adaptive immunity, engineered macrophage therapies are being developed to steer the innate response. This includes infusing macrophages pre‐polarized to a pro‐reparative M2‐like phenotype or using CAR‐M cells designed not for cytotoxicity but to deliver anti‐inflammatory cytokines locally at sites of injury, actively quenching the inflammatory cascade.

Certain cardiovascular conditions have explicit autoimmune components, for which targeted lymphodepletion strategies show promise. A paradigm is found in anti‐B‐cell maturation antigen (BCMA) CAR‐T therapy, successfully used in a case of refractory immune‐mediated necrotizing myopathy [156]. By eliminating autoantibody‐producing B‐cell lineages, this approach achieved dramatic clinical improvement and reduced pathogenic autoantibodies. This principle is translatable to cardiovascular diseases with autoimmune features, such as certain forms of myocarditis or vasculitis, where CAR‐T cells could be designed to target specific autoreactive B or T cell clones, offering a potent and specific reset of the pathogenic immune response.

The evolution of cellular immunotherapy for cardiovascular aging is marked by a shift from broad immunosuppression to targeted immunomodulation. Current strategies are highly mechanism‐driven: CAR‐T/NK/M cells directly eliminate pro‐fibrotic cells, Tregs and engineered macrophages actively resolve harmful inflammation, and targeted lymphodepletion addresses autoimmune pathogenesis. Coupled with innovative in vivo delivery platforms like mRNA‐LNPs, these approaches promise to transform the management of age‐related cardiovascular diseases by directly intervening in the specific immune dysregulations that drive pathology, moving from symptom management to targeted disease modification.

### 4.5 Other Aging‐Associated Pathologies

Organ fibrosis, a central pathological hallmark of numerous chronic aging‐related diseases, affects multiple organs including the heart, liver, lungs, and kidneys.

In pulmonary fibrosis, aberrant activation and proliferation of fibroblasts drive excessive ECM deposition. Fibroblast activation protein (FAP)‐targeted CAR‐T cells, generated using lipid nanoparticle (LNP)‐mRNA delivery systems, have shown promise in selectively eliminating hyperactivated fibroblasts [158]. Preclinical models demonstrate that FAP‐CAR‐T cells not only attenuate fibrotic lesions but also promote lung tissue regeneration [158]. Additionally, macrophage‐targeted therapies have been explored. Studies by Sang et al. revealed that modulating macrophage polarization or function can reshape the immune microenvironment, synergistically enhancing anti‐fibrotic efficacy in lung fibrosis models [159].

Renal fibrosis is exacerbated by pro‐fibrotic immune subsets such as CD4+ T cells and Th17 cells, which amplify inflammatory damage. In contrast, Tregs exhibit protective effects by suppressing immune responses and preventing ECM accumulation. Preclinical study in animal models confirm that Treg‐based cell therapy mitigates renal inflammation and reverses fibrotic remodeling [160]. Besides, aiming at the pathological hallmark of renal fibrosis — excessive accumulation of ECM, Song et al. developed a CAR‐T cell therapeutic system targeting ECM‐producing cells, including fibroblasts, pericytes, and myofibroblasts [75]. This approach alleviates multi‐organ fibrosis, improves chronic kidney disease (CKD) and its cardiovascular complications induced by various etiologies, and also provides a novel therapeutic paradigm for other fibrotic diseases. However, clinical translation remains limited, necessitating further validation of safety and long‐term efficacy in human trials.

## 5 Remaining Challenges

Immunotherapeutic approaches targeting senescent cells represent a promising avenue for addressing aging; however, their clinical application is hindered by several significant challenges.

First, issues of targeting specificity and safety remain paramount. Current investigations have yet to fully elucidate the fundamental mechanisms governing both natural aging and pathologically accelerated aging syndromes. This gap is compounded by a critical shortage of validated biomarkers suitable as therapeutic targets. Moreover, the absence of unique surface markers on senescent cells restricts the efficacy of targeted clearance strategies. For instance, in CD19‐directed CAR‐T cell therapy for SLE, preliminary clinical findings indicate transient depletion of B cells, yet the long‐term immunological outcomes remain unclear [88, 161]. Similarly, in the context of chronic organ fibrosis, although activated fibroblasts (FAP as target) and extracellular matrix‐producing cells (PDGFRβ as target) contribute to pathological remodeling, the prolonged elimination of these cells via targeted therapies may result in unintended long‐term functional impairments of the affected organs—a risk that has not been fully characterized [75, 162, 163]. These examples highlight the imperative to rigorously assess the physiological consequences of senolytic cell‐based therapies, emphasizing the need for strategic antigen selection that can effectively distinguish pathological senescent cells from their functional counterparts within homeostatic tissues.

Second, the immunosuppressive microenvironment constitutes a critical obstacle. Metabolic dysregulation within tumor microenvironments and aged tissues – such as hypoxia and lactate accumulation ‐ suppresses immune cell function. For example, CAR‐NK cells exhibit functional exhaustion within TME due to inadequate energy supply [164, 165, 166]. Furthermore, therapeutic efficacy is often limited by restricted migration and infiltration of immune cells into the inflammatory milieu of aged tissues, paralleling challenges observed in solid tumors. Additionally, the aging microenvironment demonstrates pronounced organ‐specific characteristics. In skeletal muscle, sarcopenia correlates with dysfunctional regulatory T cells and an accumulation of pro‐inflammatory macrophages within an immunosuppressive stem cell niche. In contrast, in the brain, therapeutic cell access is impeded by the blood‐brain barrier, and aged microglia frequently adopt a dysfunctional, disease‐associated phenotype that may hinder therapeutic interventions. Consequently, future advancements will likely depend on integrated strategies that simultaneously enhance immune cell resilience and functional persistence—through metabolic reprogramming and engineering to improve tissue homing and infiltration—and modulate the host microenvironment. This may involve co‐administration of metabolic modulators or stroma‐remodeling agents to surmount local barriers, thereby enabling efficient and specific clearance of senescent cells.

Thirdly, cellular senescence plays advantageous roles within specific physiological contexts. Under normal physiological conditions and during early life stages, senescent cells contribute critically to processes such as embryogenesis, wound healing, and tissue regeneration [10, 167, 168]. In these scenarios, the presence of senescent cells within the tissue microenvironment is transient, likely serving to mitigate the harmful consequences associated with their prolonged persistence. Moreover, within the tumor microenvironment, the induction of senescence in malignant cells can paradoxically inhibit neoplastic proliferation and metastatic spread [169, 170, 171], thereby diminishing overall tumor burden. Nevertheless, this beneficial regulatory mechanism becomes significantly dysregulated during tissue aging. The chronic accumulation of senescent cells in aged tissues results in sustained secretion of SASP factors, which contribute to progressive tissue dysfunction. A comprehensive understanding of this “dual nature” of senescent cells offers a strategic framework for the development of targeted therapeutic interventions. Rather than broadly suppressing the entire senescence process — which risks disrupting its essential physiological functions — therapeutic efforts should focus on selectively eliminating abnormally persistent senescent cells or effectively inhibiting their deleterious SASP. In summary, cellular senescence constitutes an evolutionarily conserved and highly dynamic cellular state. During early life and under specific physiological conditions, it operates as a tightly regulated, transient program with beneficial effects. However, in the context of organismal aging or chronic stress, due to impaired immune clearance and the sustained presence of SASP, senescence transitions into a dysfunctional driver of various pathological processes. Therefore, the future of anti‐aging cellular therapies hinges on the precise identification and targeted elimination of these detrimental senescent cells. This objective may be realized through approaches such as synthetic biology “gating” strategies, wherein therapeutic cells are engineered to initiate targeted clearance exclusively upon detection of specific signals within the aged tissue microenvironment. Such methodologies aim to substantially reduce the burden of aging‐related diseases while minimizing disruption to normal physiological functions.

Fourth, the cellular origin and functional state critically impact therapeutic outcomes. Current clinically approved cell therapies exclusively rely on autologous immune cells that undergo ex vivo genetic modification prior to reinfusion into patients. However, this approach encounters significant challenges in elderly populations due to intrinsic immunological deficits, such as T cell functional exhaustion accompanied by reduced T cell receptor (TCR) diversity [172], pro‐inflammatory polarization of macrophages [173], and dysregulation of NK cells [174]. These impairments, compounded by inter‐individual variability, collectively contribute to unpredictable therapeutic responses and inconsistent clinical outcomes. In contrast, immune cell platforms derived from healthy donors or generated via iPSCs offer three notable advantages: (1) production of functionally robust cellular products resistant to age‐associated decline, (2) consistent batch‐to‐batch quality that complies with pharmacopeial standards, and (3) scalable manufacturing processes that facilitate off‐the‐shelf availability. Nonetheless, allogeneic approaches face enduring technical challenges, particularly the necessity for complex multi‐locus genomic modifications — such as simultaneous HLA knockout, CAR integration, and suicide gene incorporation — and prolonged production timelines (4–6 weeks) required for thorough differentiation and expansion protocols.

Finally, cost and accessibility concerns cannot be overlooked. Cell‐based therapeutics represent a distinct medicinal products that differ from conventional macromolecular or small‐molecule drugs, as they are living entities [175]. Their manufacturing involves multiple critical stages, including cell harvesting, isolation, genetic modification, expansion, and cryopreservation, forming a complex technological workflow. Notably, genetic engineering and cell proliferation steps demand exceptional technical precision and process stability. Conventional production methods, which rely heavily on manual operations, exhibit suboptimal efficiency and heightened contamination risks. Moreover, even minor deviations occurring at any stage of this intricate process can lead to significant inter‐batch variability, undermining product consistency. The high manufacturing costs associated with CAR‐T therapies, coupled with the necessity for personalized production, impede widespread adoption and limit accessibility to broader patient population [176, 177, 178].

Given the complexity and heterogeneity inherent in aging mechanisms, as well as potential biological trade‐offs, intervention strategies must transcend reliance on short‐term biomarkers. It is imperative to establish a comprehensive, long‐term, multidimensional, and stratified safety assessment framework [179]. Prioritizing the implementation of such strategies in elderly populations is essential to achieve an optimal balance between lifespan extension and the avoidance of long‐term adverse effects.

## 6 Future Perspectives

The remarkable clinical success of engineered immune cell therapies in oncology has established a powerful technological and conceptual foundation. Looking ahead, the field is poised for a transformative expansion into the broader landscape of age‐related diseases. The future development of these therapies will focus on translating oncological innovations to target the shared hallmarks of aging — such as cell senescence, chronic inflammation, and tissue fibrosis — that underpin conditions like chronic disease, vascular aging, musculoskeletal disorders, metabolic syndrome and so on. This evolution will be driven by key areas: enhancing efficacy and safety through biomaterial and synthetic biology tools, advancing targeted in vivo delivery and gene editing, developing rational combinatory strategies, and directly reprogramming the aged immune system itself.

### 6.1 Biomaterials‐Assisted Cellular Immunotherapy

Biomaterials demonstrate transformative potential in advancing immunotherapies through three synergistic strategies. In immune cell expansion, 3D scaffolds (e.g., PEG/HA hydrogels) mimic native extracellular matrix properties to enhance T/NK cell proliferation [180, 181], while functionalized artificial antigen‐presenting cells (aAPCs) optimize CAR‐T expansion efficiency [180, 181]. Implantable alginate hydrogels enable localized delivery of CAR‐T cells for postoperative tumor control [181, 182]. For targeted delivery, stimuli‐responsive nano‐systems like Mn‐coordinated nanoparticles achieve tumor microenvironment‐specific release of STING agonists [183], complemented by biomimetic Mel‐SiO_2_@CCM nanoparticles demonstrating dual‐targeting capability against tumors and oncogenic bacteria [184]. Innovative administration routes are exemplified by DOCA‐based nasal vaccines overcoming mucosal barriers [185] and ferritin nanocages co‐delivering antigens/siRNA to boost DC‐T cell crosstalk [186]. Regarding immune microenvironment regulation, nanocarriers delivering SOCS1 siRNA reverse immunosuppression by restoring DC functionality [186], while injectable hydrogels loaded with checkpoint inhibitors promote T‐cell infiltration [180, 181]. Emerging approaches integrate exosome‐mimetic particles and 3D‐printed tracheal scaffolds with PEGDA‐DTT hydrogels to coordinate macrophage autophagy and T‐cell activation [182], establishing multifunctional platforms for immunomodulation and tissue regeneration. Currently approved CAR‐T therapies remain challenged by high costs and substantial safety risks. mRNA‐LNP technology offers distinct advantages, including viral vector independence, elimination of genomic integration risks, rapid manufacturing, and scalability [187]. The integration of mRNA‐LNP with CAR‐engineered immune cells represents a cornerstone of next‐generation immunotherapy. Concurrently, precisely modulating CAR‐T cell activity using intelligent biomaterials, such as hydrogels, PEGylated systems, and photo‐responsive platforms, can significantly mitigate toxicities (e.g., CRS, ICANS, and on‐target/off‐tumor effects) without compromising therapeutic efficacy [188]. These material‐driven strategies collectively address critical bottlenecks in cellular immunotherapy through precision engineering of biological interfaces.

Biomaterial‐driven immune engineering strategies described above are fundamentally centered on the precise modulation of immune cell function, enabling targeted delivery, and reshaping the local microenvironment. These principles establish a transformative framework for treating chronic diseases closely associated with aging. In the context of vascular aging and atherosclerosis, pathological processes encompass endothelial cell dysfunction, vascular calcification, and chronic inflammation. Biomimetic nanoparticles, such as membrane‐coated nanoparticles, can be engineered to target specific cell populations within activated vascular endothelium or atherosclerotic plaques, such as macrophages. These particles can deliver mRNA‐encoded anti‐inflammatory cytokines (e.g., IL‐35) or proteins mediating reverse cholesterol transport to stabilize plaques. Moreover, biomaterial scaffolds for cell delivery, including hydrogel patches, can be utilized to load and locally deliver engineered macrophages or endothelial progenitor cells endowed with anti‐inflammatory properties. This approach facilitates the repair of damaged vascular endothelium, analogous to strategies for localized CAR‐T cell delivery post‐tumor resection. Regarding musculoskeletal disorders such as osteoarthritis and osteoporosis, degenerative changes in joints and bones are accompanied by persistent inflammation and diminished regenerative capacity. Localized sustained‐release systems based on biomaterials—such as thermosensitive or shear‐thinning hydrogels—allow for direct intra‐articular injection. They enable the controlled release of kallikrein inhibitors, neutralizing antibodies targeting the senescence‐associated secretory phenotype (SASP), or growth factors that promote cartilage/bone regeneration (e.g., TGF‐β3, BMP‐2). These systems can work synergistically with engineered regulatory T cells to achieve long‐term modulation of immune imbalance within the joint cavity, thereby protecting cartilage and promoting tissue repair.

In summary, biomaterial‐assisted cellular immunotherapy is providing targeted delivery, local immune reprogramming, and structural tissue support, offering transformative strategies for combating aging‐related diseases.

### 6.2 The Integration of Synthetic Biology

Synthetic biology offers innovative technological pathways for immune cell‐based therapies, especially on engineered immune cell reprogramming and functional enhancement [189, 190].

The synthetic membrane‐integrated proteolytic receptor (SNIPR) developed by researchers employs modular segmentation to mitigate immunogenicity risks while enhancing receptor activation efficiency [191, 192]. This architecture enables CAR‐T cells to sense soluble ligands (e.g., TGF‐β, VEGF) with tumor microenvironment‐restricted activation, minimizing off‐target toxicity. Building on this framework, they refined SNIPR to detect soluble immune signals in tumor niches [192], implementing a dual‐validation mechanism (environmental trigger plus antigen recognition) for enhanced specificity. Parallel innovations include DAP12‐based synthetic antigen receptors (DAP12‐SARs) enabling multi‐antigen targeting (e.g., IL13Ra2/HER2) through orthogonal receptor design. For instance, HER2‐specific TREM1‐scaffolded receptors combined with IL13Ra2‐targeted KIR2DS2‐derived receptors demonstrate synergistic elimination of heterogeneous tumors [193]. Complementing these advances, iPSC‐derived CAR‐NK platform integrates CD70 CAR and IL15RF modules to boost tumor cytotoxicity and persistence [194].

Building upon the shared hallmarks between tumors and aged tissues—such as strong heterogeneity in surface antigen expression, a hypoxic and acidic microenvironment, and potent immunosuppression—therapeutic strategies employing dual‐response mechanisms to microenvironmental signals and antigen targets, or multi‐antigen targeting to enhance specificity and clearance efficiency, are similarly applicable to anti‐aging interventions. For instance, one could engineer CAR‐T cells or macrophages whose activation requires the simultaneous recognition of two antigens co‐expressed on senescent cells but not concurrently on healthy cells (e.g., uPAR and EGFR). This approach would significantly improve targeting specificity, preventing off‐target attacks on normal tissues expressing only one antigen (e.g., EGFR+ skin cells). Furthermore, a co‐stimulatory module controlled by hypoxia‐response elements (HREs) or pH‐sensitive promoters could be incorporated. Full activation and effector functions would be triggered only when the cells engage both target antigens and are within a hypoxic or low‐pH milieu. This ensures the therapy acts primarily on pathological senescent niches (e.g., the core of atherosclerotic plaques, fibrotic kidneys) rather than areas of physiological aging or mild inflammation.

Unlike anti‐tumor therapies where direct killing or phagocytosis is the primary objective, eliminating senescent cells need not be the first option in anti‐aging treatment. Accordingly, we can design these cells as “programmable therapeutic platforms” that dynamically output different responses based on perceived signals. For example, engineered cells can function as “microenvironment modulators.” They can be programmed to secrete decoy receptors or enzymes that degrade key pro‐inflammatory SASP factors (e.g., IL‐6, IL‐1β, MMPs), thereby dampening chronic inflammation. Simultaneously, they could express immunomodulatory cytokines (e.g., IL‐10, specific isoforms of TGF‐β) to reprogram pro‐fibrotic M2 macrophages or effector T cells toward a reparative/regulatory phenotype, addressing the tissue environment at its root.

A “scout‐clear‐repair” cascade response can also be engineered. In the initial phase, the cells secrete antibody‐based “tags” (e.g., single‐chain antibodies targeting specific senescent cell surface antigens) to label senescent cells for recognition and clearance by the endogenous immune system (e.g., macrophages), without direct killing. If inflammatory or fibrotic signals persist after tagging, the cells can switch to a direct clearance mode (e.g., releasing granzyme B). Finally, upon completing the clearance task, a tissue repair program can be initiated, involving the secretion of growth factors (e.g., VEGF, FGF2) or matrix‐remodeling enzymes (e.g., MMPs) to promote healthy tissue regeneration.

Given the chronic nature of aging‐related diseases, long‐term and controllable treatment is essential. To this end, drug‐inducible or small molecule‐controlled switches can be integrated—such as gene expression systems activated or silenced by oral small molecules (e.g., rapamycin, tamoxifen). This allows clinicians to precisely activate the engineered cells' functions during therapeutic windows (e.g., acute fibrotic exacerbation) and maintain them in a resting state during maintenance phases, thereby minimizing long‐term side effects. As with cancer therapies, fail‐safe mechanisms must be incorporated, such as suicide switches (e.g., inducible caspase9). Furthermore, self‐limiting programs can be designed to activate automatically upon completion of a specific task (e.g., after clearing a predefined number of senescent cells) or in case of abnormal proliferation, ensuring the therapy is temporally and spatially confined. This integrated, logic‐gated approach represents a sophisticated and promising frontier in the development of next‐generation, precision anti‐aging therapies.

### 6.3 Targeted Delivery

Targeted delivery strategies significantly enhance the efficacy and safety of cellular immunotherapy through precision control of therapeutic agent transport. Recent advances integrating nanotechnology, bioengineering, and materials science have yielded innovative systems for tumor immunotherapy, infectious diseases, and genetic disorders.

Targeted delivery systems represent a paradigm shift in therapeutic precision, orchestrating spatiotemporal control over immunomodulatory agents through three principal technological frameworks. Nanoparticle platforms dominate this field, exemplified by lipid nanoparticles (LNPs) employing PEGylation to prolong circulation and exploit enhanced permeability retention (EPR) effects for tumor targeting [195, 196]. Inorganic counterparts like manganese‐based nano‐systems (e.g., LMMH) synergize cGAS‐STING pathway activation via Mn^2^^+^ release with chemotherapeutic interference of tumor metabolism [183, 197, 198], while stimuli‐responsive polymeric carriers (e.g., PLGA) achieve precision payload release through pH/redox‐sensitive designs [196, 199]. Complementing synthetic vectors, bioengineered bacterial carriers (e.g., BactPac) function as in situ bioproducers of gene‐editing tools, leveraging receptor‐mediated tropism (e.g., CD29 targeting) to enhance tumor specificity. Concurrently, nanovector‐encapsulated oncolytic virotherapeutics bypass immunogenicity concerns while inducing immunogenic cell death [195, 196]. Emerging physical approaches like NanoFLUID electroporation patches demonstrate transformative potential, amplifying intracellular delivery efficiency by orders of magnitude through “nanochannel‐microelectrode” architectures with >95% cell viability in preclinical models [200]. These multimodal strategies collectively redefine therapeutic windows by integrating material intelligence with biological specificity.

To address the challenges posed by the disseminated nature of lesions, target heterogeneity, and the need for long‐term, controllable intervention in aging‐related diseases, therapeutic cells must be engineered or integrated with biomaterial carriers to endow them with active targeting capabilities.

Utilizing synthetic biology, therapeutic cells—such as regulatory T cells, or engineered macrophages — can be equipped with surface‐expressed homing receptors that target molecules highly expressed in the aged tissue microenvironment. For instance, receptors targeting Vascular Cell Adhesion Molecule‐1 (VCAM‐1), which is upregulated on inflamed/senescent endothelium, can be deployed. Meanwhile, genetic engineering can be employed to overexpress receptors on therapeutic cells that match chemokines secreted by senescent lesions (e.g., CCL2, CXCL12). This enables the cells to actively migrate along chemotactic gradients to the target sites, a strategy particularly applicable to chronic inflammation‐driven diseases like atherosclerosis or osteoarthritis. Enhanced physical targeting can be realized by conjugating engineered cell with magnetic nanoparticles which allows for guided enrichment in specific organs (e.g., kidneys, liver) or joints via external magnetic fields. This approach significantly increases local cell concentration while minimizing systemic distribution.

Once therapeutic cells reach the lesion, their efficacy would be transient without effective retention. Targeted delivery materials can provide a temporary or long‐term “niche.” Injectable Bioactive Scaffolds: Encapsulating therapeutic cells within injectable hydrogels or microcarriers allows for direct delivery to target tissues (e.g., joint cavity, peri‐infarct myocardium, subcutaneous adipose tissue). These materials not only provide physical support but, through their matrix components (e.g., hyaluronic acid, decellularized matrix) and loaded biological cues (e.g., integrin‐binding peptides, growth factors that maintain an undifferentiated or pro‐reparative cell phenotype), can mimic the native microenvironment to support cell survival, proliferation, and sustained secretion of therapeutic factors. Local Sustained Release and Protection: The delivery system can be co‐loaded with nutritional factors and anti‐apoptotic agents to protect the therapeutic cells within the hostile aged microenvironment (characterized by high oxidative stress and low nutrients). Simultaneously, the system can enable the controlled release of signaling molecules (e.g., cytokines, small molecule agonists) required to dynamically guide cell behavior within the lesion.

Future development will focus on creating more intelligent, biocompatible, and host‐integrative “cell‐material hybrid systems.” This strategy not only holds significant promise for enhancing the safety and efficacy of cell therapies targeting chronic conditions like kidney disease and vascular aging but may also catalyze the development of long‐term implantable, on‐demand activatable “living drugs.” This would lay a solid foundation for achieving preventive, personalized, and reversible anti‐aging interventions.

### 6.4 In Situ Gene Editing

In situ gene editing technologies are revolutionizing cell‐based immunotherapies by enabling direct in vivo genetic modifications [111, 201], circumventing traditional ex vivo processing limitations. The enGager system enhances large‐fragment integration (e.g., CAR genes) via Cas9 fused with single‐strand DNA‐binding motifs (e.g., FECO), achieving 33.4% CAR integration rates in T cells, a sixfold improvement achieved [202]. For T‐cell engineering, viral/nanocarrier‐mediated CAR gene delivery enables in vivo generation of functional CAR‐T cells. SEED‐Selection streamlines manufacturing through one‐step immunomagnetic negative selection, yielding 90% purity in multiplex‐edited (TRAC/B2M/CD4) T cells [203]. CRISPR‐mediated PD‐1 knockout via conductive hydrogel delivery systems achieves 80% melanoma recurrence suppression through lymph node‐targeted editing [204].

Beyond T‐cells, MAGE platform employs poly(β‐amino ester) nanoparticles to deliver CasRx, silencing macrophage “don't eat me” signals (SIRPα/Sig10) and reducing tumor volume by > 40% [202, 205]. one research employed low‐immunogenic lipid nanoparticles to deliver PD‐L1 mRNA, programming the in vivo generation of tolerogenic antigen‐presenting cells (tol‐APCs). These tol‐APCs selectively inhibit pathogenic T cells and expand regulatory T cells [143]. This approach showed remarkable efficacy in rheumatoid arthritis and ulcerative colitis models, with a single treatment costing merely 1% of conventional therapies. For hematopoietic stem cells, epitope‐editing strategy using prime editors introduces CD123 mutations, shielding HSPCs from CAR‐T attack while preserving functionality [206]. In addition, RNA therapeutics are emerging as a novel platform for slowing aging and treating age‐related diseases. They encompass six major strategies: RNA activation, mRNA supplementation, RNA interference, ASOs, aptamers, and CRISPR‐Cas13 [207]. These approaches can precisely upregulate protective genes or downregulate pathogenic ones, and efficacy has been demonstrated in multiple phase I‐III clinical trials targeting Alzheimer's disease, hypercholesterolemia, osteoporosis, and muscular atrophy [207]. These precision tools collectively advance next‐generation therapies by balancing efficacy with safety across diverse immune cell types.

In situ gene editing represents a more direct and generalized paradigm of “cell therapy,” which aligns particularly well with the core objectives of anti‐aging interventions. It aims to achieve precise and potentially reversible functional modulation within complex aged tissues, rather than employing indiscriminate clearance. For instance, leveraging CRISPR interference (CRISPRi) technology allows for the targeted transcriptional repression of key SASP regulator genes (e.g., components of the NF‐κB or p38MAPK pathways), thereby shutting down the excessive secretion of pro‐inflammatory factors. Alternatively, CRISPR activation (CRISPRa) can be utilized to upregulate the expression of regenerative genes (e.g., YAP, NOTCH) or rejuvenation factors (e.g., KLOTHO) in hematopoietic stem or progenitor cells, enhancing their self‐renewal and differentiation capacity. The strategy is not to kill senescent cells but to edit their genomes, converting them from a detrimental pro‐inflammatory phenotype into a beneficial regenerative or quiescent state.

### 6.5 Combinative Therapeutic Strategies

The synergy between immune cell therapy and conventional treatments is exemplified by chemotherapy or radiotherapy inducing immunogenic cell death (ICD) in tumor cells, thereby releasing tumor antigens to amplify immune recognition. For instance, a localized therapy combined with immunotherapy (LRT‐IO) achieved long‐term complete remission in 46% of advanced hepatocellular carcinoma patients, with survival rates approaching those of curative surgery [208]. Furthermore, the combination of CAR‐T cell therapy and immune checkpoint inhibitors — such as uPAR‐targeted CAR‐T cells paired with PD‐1 inhibitors — demonstrated marked suppression of tumor recurrence in gastric cancer models by simultaneously eliminating tumor cells and reversing T‐cell exhaustion [209]. Novel combinatorial immunotherapies have also shown breakthroughs; for example, cDC1 dendritic cell vaccines combined with CTLA‐4 or PD‐1 inhibitors achieved 100% recurrence inhibition in melanoma and colon cancer models [210].

For HIV treatment, the integration of gene editing and immunotherapy demonstrates promise: A study engineered CD8 T cells with synNotch receptors to co‐secrete broadly neutralizing antibodies (bNAbs) and bispecific T‐cell engagers (BiTEs), achieving dual effects of neutralizing free viruses and eliminating infected cells in vitro, alongside sustained viral suppression [123]. Additionally, CAR‐T therapy combined with antiretroviral drugs enhances the clearance of latently infected cells, advancing progress toward HIV functional cure [123].

Immunomodulatory combinations have also progressed, such as the chimeric peptide E16‐uPA24, which targets uPAR on senescent cells and activates the mGluR5 receptor on NK cells, significantly improving NK cell‐mediated clearance of senescent cells and ameliorating tissue microenvironments in liver and pulmonary fibrosis models [28].

Artificial intelligence (AI) is driving transformative advances in anti‐aging research. The structure of a CAR is a decisive determinant of its function, with the affinity, specificity, and stability of its antigen‐binding domain (scFv) is particularly critical. Traditional antibody discovery relies on animal immunization or screening of large phage display libraries, processes that are time‑consuming and labor‑intensive. The Chai‑2 model directly enables de novo design of full‑length therapeutic monoclonal antibodies, with up to 86% of its designed antibodies meeting drug‑lead standards in developability metrics such as stability, expressibility, and low aggregation. This suggests that in the future, AI could rapidly generate optimal scFv sequences against specific aging‑related targets for direct use in CAR construction, greatly shortening the candidate‑drug discovery cycle. AI systems such as DeepTarget integrate large‑scale drug and genetic screening data to predict the mechanism of action and potential targets of small molecules or antibodies within specific cellular contexts. This “context‑specific” target‑prediction capability provides important guidance for designing CARs with differential affinities that distinguish between highly expressed senescence‑associated antigens and low‑expression normal‑tissue antigens, thereby reducing on‑target/off‑tissue toxicity [211]. AI can also extract latent features from high‑dimensional data—including radiomics, pathomics, genomics, and multi‑omics—to build predictive models for non‑invasively quantifying features of the tumor or senescent tissue microenvironment, enabling early prediction of the potential efficacy of CAR‑T or TCR‑T cell therapies [212]. The C2S‑Scale model treats single‑cell transcriptomic data as “sentences.” By training large‑parameter language models, it not only interprets individual cell states but also predicts the systemic impact of external perturbations, such as drugs, on cell‑population behavior [213]. This technology can be used for in‑silico screening of candidate molecules that enhance CAR‑T cell persistence or reverse exhaustion, or for predicting the risk of severe adverse effects like cytokine release syndrome (CRS), allowing risk stratification and intervention planning prior to therapy. For highly heterogeneous cancers and senescent tissues, identifying truly immunogenic and specific targets remains a core challenge. Through unsupervised learning, AI can uncover novel, human‑unanticipated senescence‑associated patterns and biomarker combinations within vast datasets. For these personalized targets, AI‑based virtual screening or generative chemistry can design entirely new intervention molecules. Therapeutic proteins, including scFvs used in CAR design, may themselves elicit unwanted anti‑drug antibody (ADA) responses. The T‑SCAPE model—a multi‑domain deep‑learning framework—leverages adversarial domain adaptation to evaluate and optimize scFv sequences at the CAR design stage [214], reducing the risk of rapid clearance of infused CAR‑T cells by the patient's immune system, thereby improving therapeutic safety and durability.

### 6.6 Rejuvenation of Aged Immune System

The core mechanisms of immune‐senescence encompass thymic involution, myeloid‐biased differentiation of HSCs, lymphocytes functional impairment, and systemic aging driven by the inflammatory microenvironment. Potential intervention strategies target three key tiers. Reconstituting immune‐generative sources: Restore thymic output via exogenous RANKL or FOXN1 gene therapy to rejuvenate the thymic niche [215, 216], combined with p38 MAPK inhibition or antibody‐mediated clearance of CD150^+^ myeloid‐biased HSCs to reverse HSC aging [31]; Restoring lymphocytes functionality: Lower the activation threshold of naïve CD4^+^ T cells using miR‐181a mimetics or DUSP6 inhibitors (e.g., BCI) [217], alleviate epigenetically locked exhaustion in CD8^+^ T cells via demethylating agents (e.g., decitabine) [218], and enhance antiviral/antitumor cytotoxicity in aged CD8^+^ T cells through PD‐1 blockade [219]; Eliminating systemic senescence drivers: Deploy senolytics (e.g., uPAR‐targeting CAR‐T cells) to clear p16^INK4a^‐positive senescent cells [220], inhibit pro‐geronic EVs secretion from aged bone marrow macrophages (BMMs) using PPARα agonists (e.g., fenofibrate), and suppress inflammatory pathways via metabolic reprogramming (e.g., metformin/rapamycin/caloric restriction) [221].

This tripartite intervention paradigm (elimination–restoration–reconstitution) aims to rebuild T‐cell diversity, enhance immune surveillance, and ultimately reverse age‐associated vulnerabilities — including infection/cancer susceptibility, impaired vaccine efficacy, and senescence‐related pathologies — providing a clinically actionable framework for personalized immune rejuvenation.

## 7 Conclusion

The primary objective of anti‐aging interventions is to decelerate fundamental aging processes and thereby mitigate a spectrum of age‐related pathologies, including cancer, cardiovascular disorders, neurodegenerative diseases, and autoimmune conditions. Research on pharmacological and methodological strategies has advanced rapidly, yielding promising outcomes in preclinical models. However, their translation into human clinical trials remains constrained by concerns regarding potential side effects [179, 222, 223].

Aging is driven by multifaceted and interconnected mechanisms, among which the direct causal relationship between immunosenescence and organismal aging requires further elucidation. Building upon the transformative success of cellular immunotherapies in oncology, this paradigm has been strategically extended to target aging and its associated diseases [132]. A growing body of evidence indicates that immune cell‐based therapies can not only selectively eliminate senescent cells and ameliorate the associated pro‐inflammatory microenvironment but also contribute to the restoration of tissue homeostasis and delay the progression of multiple age‐related conditions.

Nevertheless, similar to the challenges encountered in cancer immunotherapy, anti‐aging cellular immunotherapies face significant translational hurdles. These include a limited repertoire of highly specific targets, the presence of immunosuppressive barriers in aged tissues, high costs of development and manufacturing, and the current absence of precise, standardized metrics for evaluating therapeutic efficacy in the context of aging. Despite these obstacles, concurrent breakthroughs in biomaterials, synthetic biology, and in vivo engineering technologies underscore the field's considerable transformative potential. Future progress will likely depend on the development of personalized therapeutic strategies [224] and deeper multidisciplinary integration, exemplified by AI‐driven synthetic biology. Such convergence is anticipated to propel the field toward precision geroscience, ultimately aiming to enhance population‐wide healthspan and longevity.

## Author Contributions

J.H.G. and L.Y.S. conceived the topic, drew up the outline, wrote and revised the original draft. All authors reviewed and edited the draft. J.H.G. and L.Y.S. provided research funding for the project. J.H.G produced the figures and tables of the manuscript. All authors read and approved the final manuscript.

## Funding

This work was supported by the National Natural Scientific Funds (822770014 and 81991523), and Zhejiang Shuren University Startup Foundation (KXY0224104)

## Conflicts of Interest

The authors declare no conflicts of interest.

## Ethics Declarations–Ethics Approval and Consent to Participate

The authors have nothing to report.

## Data Availability

No datasets were generated or analyzed during the current study.
